# Dynamic emotion intensity estimation from physiological signals facilitating interpretation via appraisal theory

**DOI:** 10.1371/journal.pone.0315929

**Published:** 2025-01-24

**Authors:** Isabel Barradas, Reinhard Tschiesner, Angelika Peer

**Affiliations:** 1 Faculty of Engineering, Free University of Bozen-Bolzano, Bolzano, South Tyrol, Italy; 2 Faculty of Education, Free University of Bozen-Bolzano, Brixen, South Tyrol, Italy; National Institute of Technology, India (Institute of National Importance), INDIA

## Abstract

Appraisal models, such as the Scherer’s Component Process Model (CPM), represent an elegant framework for the interpretation of emotion processes, advocating for computational models that capture emotion dynamics. Today’s emotion recognition research, however, typically classifies discrete qualities or categorised dimensions, neglecting the dynamic nature of emotional processes and thus limiting interpretability based on appraisal theory. In our research, we estimate emotion intensity from multiple physiological features associated to the CPM’s neurophysiological component using dynamical models with the aim of bringing insights into the relationship between physiological dynamics and perceived emotion intensity. To this end, we employ nonlinear autoregressive exogeneous (NARX) models, as their parameters can be interpreted within the CPM. In our experiment, emotions of varying intensities are induced for three distinct qualities while physiological signals are measured, and participants assess their subjective feeling in real time. Using data-extracted physiological features, we train intrasubject and intersubject intensity models using a genetic algorithm, which outperform traditional sliding-window linear regression, providing a robust basis for interpretation. The NARX model parameters obtained, interpreted by appraisal theory, indicate consistent heart rate parameters in the intersubject models, suggesting a large temporal contribution that aligns with the CPM-predicted changes.

## Introduction

Nowadays, we live in a world characterised by an increasing number of intelligent technological devices. Consequently, we spend more and more of our time interacting with these devices, both in professional and social contexts. As users, we want this interaction to be as natural as possible. The ability to recognise emotions is hereby considered an important ingredient, given that, with such ability, machines will be able to adapt their behaviour accordingly—just like humans [1, 2].

Developments in emotion appraisal models brought to light the dynamic nature of emotions [3] and this theoretical knowledge can be applied to inspire the design of new experiments and the development of emotion models. So far, however, most studies have applied only static machine learning approaches (such as traditional statistical methods like support vector machines [4, 5] and *K*-nearest neighbours [5, 6], or deep learning (DL) approaches like convolution neural networks as in [7, 8]) to detect an affective state dominating over a whole time window. While useful, these models cannot really capture the dynamic nature of emotional processes. According to appraisal models, namely, an emotion is defined as *an emergent, dynamic process, initiated by the individual’s subjective appraisal of events* [9]. This appraisal is essential to unleash the emotional episode and implies an assessment of how an event influences the individual’s well-being. Besides its essential role to trigger an emotion, the appraisal also determines the quality and intensity of the action tendency, physiological responses, behaviour, and feelings [10].

Thus, in this work, we consider the Scherer’s Component Process Model (CPM) [9, 11], in which appraisal is considered a process in time. In the scope of the CPM, an emotion follows the evaluation of an external or internal event (stimulus) that is relevant to major concerns of the organism, and encompasses interrelated, synchronised changes in the states of all or most of five organismic subsystems [12]. These subsystems are also called components, and they are: the cognitive component, which includes the multilevel appraisal; the neurophysiological component, which represents the bodily reactions; the motivation component, which indicates the action tendencies; the motor expression component, described by the facial and vocal expressions; and finally, the subjective feeling, which represents the affective state [13]. The subjective feeling is characterised by its quality, intensity, and duration [11].

The components of the CPM are influenced according to the outcome of a set of sequential criteria called “stimulus evaluation checks” (SECs). The outcomes of the SECs are always subjective to the individual’s perception of the event [12]. With this in mind, an emotion induction experiment can be built with stimuli (events), expecting the different components to be influenced accordingly. Different components contain information from different signals, and even within the same component different signals can be affected differently (based on the SECs’ outcomes), which favours a multimodal approach. Such SECs-resulting physiological changes can be captured through the noninvasive recording of electrophysiological signals. Actually, electrophysiological signals represent a powerful tool in the field of emotion recognition, since they are able to capture spontaneous information continuously, while not being constrained by social protocols [7, 14, 15]. Since the acquisition of certain physiological signals is relatively simple to perform with noninvasive sensors, these signals are able to be monitored in real time. In this work, we focus on cardiovascular, electrodermal, and respiratory measures because they give comprehensive and complementary information from the autonomic (The autonomic nervous system is the branch of the peripheral nervous system that regulates involuntary body functions (heartbeat, digestion, breathing, etc.)). emotional response [8, 16, 17].

Although appraisal models hold the potential to serve as a framework for emotion recognition studies, this is still quite uncommon in the literature, see also Table 1. Nevertheless, Somarathna *et al*. (2022) [18] conducted a study in which 20 discrete emotion terms from the GEW [19] were classified by using information related to all the different components of the CPM (even if separately), which makes it a pioneer investigation. For most components, these inputs were the answers to the CoreGRID [20]—an instrument developed to assess the different CPM components, namely the appraisal, the action tendency, the expression, the bodily reaction, and the subjective feeling. In our perspective, this study is particularly relevant because, for the physiological component, they achieved better results when also including the information from several biosignals (galvanic skin response, heart rate, blood volume pulse and skin temperature) as inputs of their models—proving that the objective measure of physiological signals is more accurate than a subjective assessment. While in the work of Somarathna *et al*. (2022) [18], the addition of physiological signals—used as an objective measurement of the neurophysiological component of the CPM rather than more-subjective responses related to the same component in the CoreGRID—aimed to improve performance, in our work we take advantage of the CPM for interpretability purposes.

**Table 1:** Comparison of related work on emotion recognition approaches, detailing emotion dimensions, model inputs, recognition methods, and CPM usage. The mentioned model inputs are galvanic skin response (GSR), CoreGRID items, BVP (blood volume pulse), HR (heart rate), skin temperature (SKT), electromyography (EMG), facial expressions, voice, and respiration (RSP) signals.

Study	Emotion dimension	Model inputs	Emotion recognition		Use of CPM	
			Classification	Regression	Performance	Interpretation
Jenke & Peer (2018) [14]	Intensity	GSR	–	✓	–	✓
Somarathna *et al*. (2022) [18]	Quality	51 CoreGRID items, BVP, GSR, HR, SKT, EMG	✓	–	✓	–
Saxena *et al*. (2022) [21]	Intensity	Facial expressions	✓	–	–	–
Rajendran *et al*. (2023) [22]	Intensity	Voice	✓	–	–	–
Our work (2024)	Intensity	GSR, HR, RSP	–	✓	–	✓

Taking into consideration these different goals of improving performance versus developing a model that facilitates interpretability, Jenke & Peer (2018) [14] were pioneers in modelling emotion dynamics to facilitate interpretation through the CPM. Unlike most studies that focus on classifying emotion qualities or discrete emotion categories, they predicted emotion intensity—a dimension that has its importance in describing an emotional experience, as also acknowledged in Saxena *et al*. (2022) [21], one of the few works in the literature dealing with intensity recognition. In Jenke & Peer (2018), a dynamic model was developed to estimate intensity from galvanic skin response (GSR) signals. This model is based on a cognitive architecture to understand the influence of SECs in the subjectively felt intensity. However, only an indicator of the neurophysiological component of emotion was considered, while this component also encompasses other responses related to the ANS, not just skin conductance. This represented a limitation since the dynamic model could not estimate all SECs’ changes. In this sense, there was a need for a study that incorporated additional information from the neurophysiological component, by including other signals reporting ANS activity. To gain a better understanding of the SECs’ contributions, and as mentioned above, we complement the electrodermal information with cardiovascular and respiratory measures.

More specifically, in this study we show that the addition of physiological features as extra information of the CPM neurophysiological component for intensity prediction is important not just to achieve better results, but also for a better comprehension of emotional processes. To achieve so, we employ a dynamic multimodal approach: nonlinear autoregressive exogenous (NARX) models, which are models that relate the current value of a time series to both its past values as well as to the past and current values of exogenous (external) variables. Here, features are combined as different exogenous variables to predict intensity for distinct emotion qualities (Happiness/Joy, Disappointment/Regret, and Worry/Fear). We also compare the relevance of features in the context of emotion prediction for the different investigated qualities, analysing it from a CPM perspective for their different profiles (information about different quality profiles can be found, for instance, in Scherer (2001) [23]).

Further, while combining signals increases the complexity of a system, the incorporation of various physiological components also brings complementary information important for a comprehensive understanding of emotional episodes [16, 24], as different physiological components can be triggered by different SECs. Thus, the presented dynamic multimodal approach aligns better with emotion theory, enhancing not only results, but especially results’ interpretability. Unlike deep learning (DL) approaches, NARX-based multimodal modelling is namely capable of modelling emotion dynamics, as well as of obtaining more insights into dynamical emotional processes, the respective role of different physiological features, and their interaction.

The remainder of the paper is structured as follows: First, the methodology adopted is explained, which includes the architecture of the used dynamic model, the setup and design of subject experiments and data collection, and the data processing as well as emotion estimation; then the evaluation procedure and results are presented followed by an overall discussion about dynamic intensity estimation and interpretation; and finally conclusions and future directions are provided.

## Methodology

### Models

#### NARX dynamical models

Within the field of system identification [25], nonlinear autoregressive exogenous (NARX) models are often used to model discrete-time nonlinear systems. This type of models explains the output, *y*, through its past values as well as the current and past values of the input *u*. In this case, *u* is considered the exogenous variable of the model. It can be described as follows:

$$\begin{array}{c} y\left(t\right)=f\left(y\left(t-1\right),\ldots ,y\left(t-n_{y}\right),u\left(t\right),u\left(t-1\right),\ldots ,u\left(t-n_{u}\right)\right)+e\left(t\right), \end{array}$$ (1)

where *t* is the discrete time-index, *u*(*t*) the input, *y*(*t*) the output, *e*(*t*) the equation error, *n*_*y*_ and *n*_*u*_ the maximum lags for the system output and input, respectively, and *f* a multiple-input, single-output, nonlinear function.

With this model, it is possible to introduce an input delay; if *n*_*k*_ represents this delay, the input **z**(*t*) is given by:

$$\begin{array}{c} \text{z}\left(t\right)=[y\left(t-1\right),\ldots ,y\left(t-n_{y}\right),u\left(t-n_{k}\right),u\left(t-n_{k}-1\right),\ldots ,u\left(t-n_{k}-n_{u}\right)]. \end{array}$$ (2)

In these equations, just linear inputs are considered. Nonetheless, the order of these models can be increased by the introduction of polynomial regressors, which is important for emotion prediction from physiological signals due to their nonlinear character [9, 26]. This will be explained in more detail immediately after, in Multimodal approach.

Here, the output of the NARX models is estimated through a combination of linear weights, an offset and a nonlinear function (containing wavelet unit functions that operate on a radial combination of inputs). This is done through the following relationship:

$$\begin{array}{c} y\left(t\right)=y_{0}+{\left(\text{z}\left(t\right)-\bar{\text{z}}\right)}^{T}\text{Pl}+W\left(\text{z}\left(t\right)\right)+S\left(\text{z}\left(t\right)\right), \end{array}$$ (3)

in which **z**(*t*) is the vector of regressors with mean $\bar{z}$, *y*_0_ the output offset (a scalar), **P** a projection matrix, **l** a vector of weights, and *W*(**z**) and *S*(**z**) the nonlinear functions of the wavelet network. *W*(*z*) is a sum of dilated and translated wavelets, *S*(**z**) is a sum of dilated and translated scaling functions.

#### Multimodal approach

We use NARX models to predict changes in emotion intensity (model output *y*) over a fixed emotion quality, considering physiological features as exogenous inputs *u*. These models are not just more informative than static models from a prediction point of view, but also allow for a better interpretation of emotional processes.

In Barradas *et al*. (2022) [27], after extracting distinct physiological features from galvanic skin response, heart rate and respiration signals, we adopted NARX models with a single exogenous variable, *i.e*., each physiological feature was assessed separately. Here, we aim for an extended version in which different exogenous variables are combined to better understand their interrelation and dependencies.

We consider linear regressors of the intensity *y* and the different physiological features *u*, polynomial (quadratic) regressors of the physiological features *u*, and the existence of delays in the physiological features *u* with respect to *y*. The amount of linear and polynomial regressors for a specific physiological feature is always the same. Mathematically, this means that the output *y*(*t*) is predicted by:

$$\begin{array}{ll} y(t) & =f(y(t-1),...,y(t-n_{y}),u_{1}(t-n_{k_{1}}),u_{1}(t-n_{k_{1}}-1),...,\\ & u_{1}(t-n_{k_{1}}-n_{u_{1}}),u_{1}{\left(t-n_{k_{1}}\right)}^{2},u_{1}{\left(t-n_{k_{1}}-1\right)}^{2},...,u_{1}{\left(t-n_{k_{1}}-n_{u_{1}}\right)}^{2},\\ & ...,u_{m}(t-n_{k_{m}}),u_{m}(t-n_{k_{m}}-1),...,u_{m}(t-n_{k_{m}}-n_{u_{m}}),u_{m}{\left(t-n_{k_{m}}\right)}^{2},\\ & u_{m}{\left(t-n_{k_{m}}-1\right)}^{2},...,u_{m}{\left(t-n_{k_{m}}-n_{u_{m}}\right)}^{2})+e(t), \end{array}$$ (4)

with *m* representing the maximum amount of combined physiological features as exogeneous inputs.

### Experiment and data collection

The dataset used in this paper is an extended version of the one introduced in Barradas *et al*. (2022) [27], now with a higher number of subjects and described in more detail.

#### Participants

The experiment was conducted with 21 healthy individuals (12 males and 9 females) aged between 19 and 43 (*M* = 29.9, *SD* = 6.3). Subjects were screened with questionnaires to exclude participants with any sign of somatization, obsessive-compulsive disorder, interpersonal sensitivity, depression, anxiety, hostility, phobic anxiety, paranoid ideation, and psychoticism (Brief Symptom Checklist [28]) as well as posttraumatic stress disorders. The questionnaires were made available in three different languages (English, Italian, and German) and thus, to ensure the validity of responses, only participants with these languages as mother tongue were accepted. Ethical approval was obtained from the Research Ethics Committee of the Free University of Bozen-Bolzano (Approval Number: DEM Cod 2020_01). Participants provided written informed consent and, at any point of the procedure, they could inform the researchers their desire to stop the experiment. The recruitment process took place between 1st of June of 2021 and 5th of May of 2023.

#### Experimental design

In this experiment, different emotions were elicited with pictures of the IAPS database [29]. All participants watched the same 96 pictures, even if in a randomised order as explained over the next paragraphs.

Since the intention was to investigate intensity profiles for different emotion qualities, we selected the following qualities: Happiness/Joy (Q1) (positive valence, high control), Disappointment/Regret (Q2) (negative valence, high control), and Worry/Fear (Q3) (negative valence, low control). Those qualities were chosen considering the quadrants of the Geneva Emotion Wheel (GEW (Instrument designed to distribute emotions according to their valence and control (dimensions directly related to the appraisal process) to describe the own subjective feeling according to emotion quality and intensity. The GEW presents some advantages over classical emotion assessment tools (*e.g*., the Self-Assessment Manikin test [30]) because its labels are closer to our communication style, and the bi-dimensional structure helps to locate the discrete emotions on the instrument. Being more intuitive than other instruments, the GEW facilitates the emotion assessment in real time, which aligns with our desired application.) [19] based on two criteria: maximise their differences in terms of valence and control, and the number of available pictures for each quadrant of the GEW. For better understanding with a visual representation, consult Sacharin *et al*. (2012) [19] or Barradas *et al*. (2022) [27].

**Fig 1:**
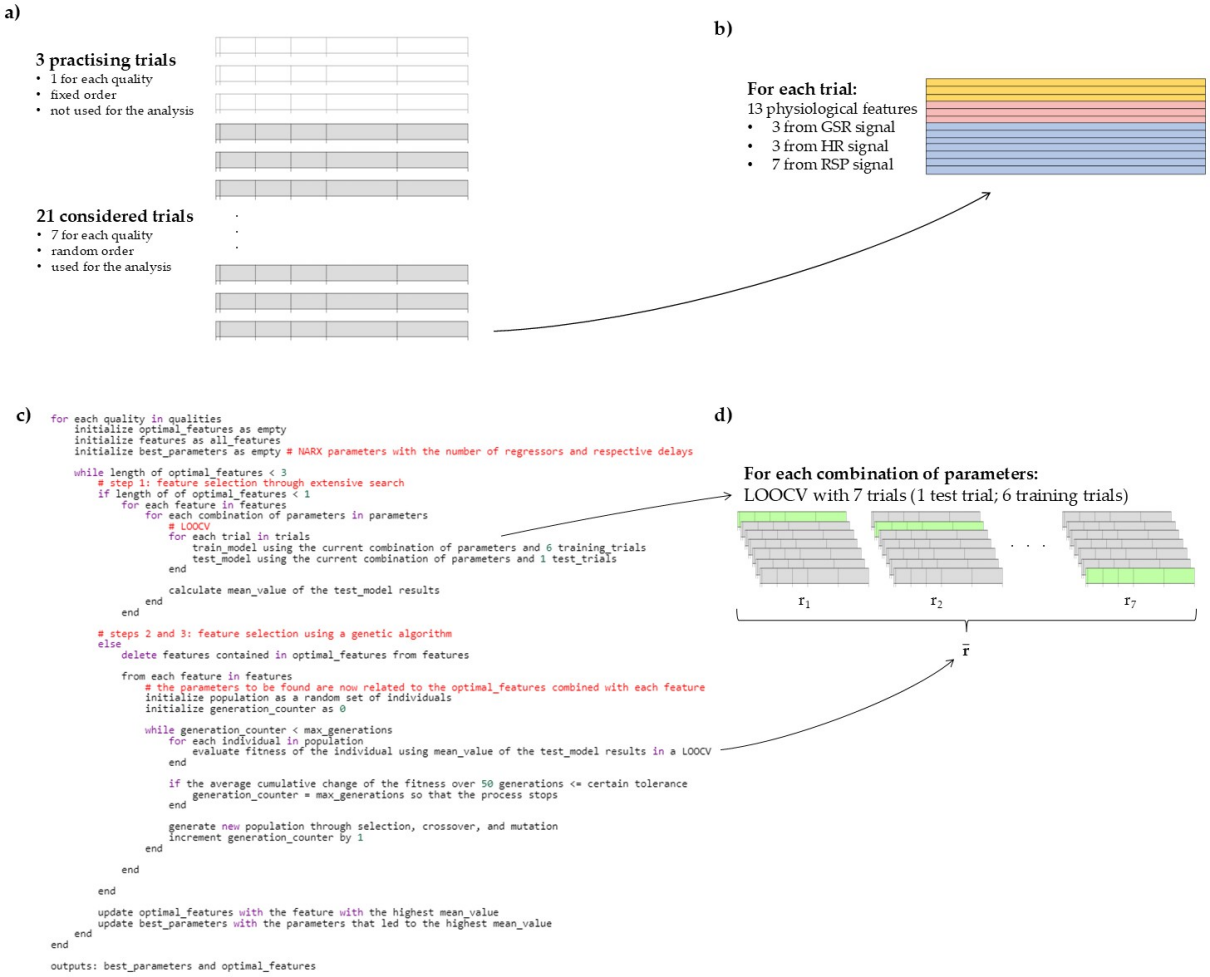
Different steps of the experiment and data processing. (**a**) The experiment consists of 24 trials corresponding to the 3 different qualities, from which 3 are practising trials (in white) and not considered for the analysis and 21 remaining trials (in grey) that are the ones used in the analysis (7 of each quality). (**b**) During the experiment, galvanic skin response, heart rate and respiration signals are collected. For each trial, 13 continuous features are extracted from these 3 physiological signals. (**c**) Pseucode explaining the intensity prediction using intrasubject NARX models, the parameters’ optimization as well as the sequential feature selection. (**d**) The models were tested with a leave-one-out cross-validation (LOOCV). This means that, for each iteration (see the lines “for each trial in trials” in the pseudocode), 6 trials were used as training trials and the remaining one as test trial. We then considered the mean correlation across iterations as the result of each feature/feature combination and each set of parameters.

This experiment had 8 trials for each emotion quality, resulting in a total of 24 trials. The order of the first 3 trials (1 for each emotion quality) was fixed, since they were meant for the participants to get familiar with the task; the remaining 21 trials were then used for the analysis, and were presented in a random order. A schematic representation is contained in Fig 1(a).

**Fig 2:**
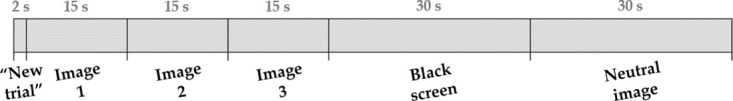
Representation of the emotion elicitation protocol. Each trial contained a message informing about the beginning of the new trial, three images meant to induce emotions of different intensities, a black screen, and a neutral image.

Each trial had a fixed quality, while the intensity of the stimuli could change over the trial. The structure of each trial (see Fig 2) was the following: a message with the information “New trial” (exhibited for 2 seconds), images representing the same quality but allowed to have different intensities (exhibited in a random order for 15 seconds each), a black screen (exhibited for 30 seconds), and a neutral image (exhibited for 30 seconds). The last two stimuli were present to help participants reaching a neutral state before the next trial. The entire experiment had an approximate duration of 43 minutes.

#### Experimental setup and procedure

The stimuli were displayed on a monitor in front of the participants. During the exhibition of images and recording of signals, participants provided real-time information about their perceived emotion with a polar device containing the GEW [19]. More specifically, participants used a knob to select quality (angle of the GEW) and intensity (proximity to the border of the GEW). Those were measured by the resistance of a rotary and a linear potentiometer, respectively, that were connected to the laptop through an Arduino UNO.

Since the quality was fixed over each trial, participants were supposed to fix their felt quality at the beginning of the first picture of the trial and, after that, just adjust the knob according to their subjectively felt intensity.

**Fig 3:**
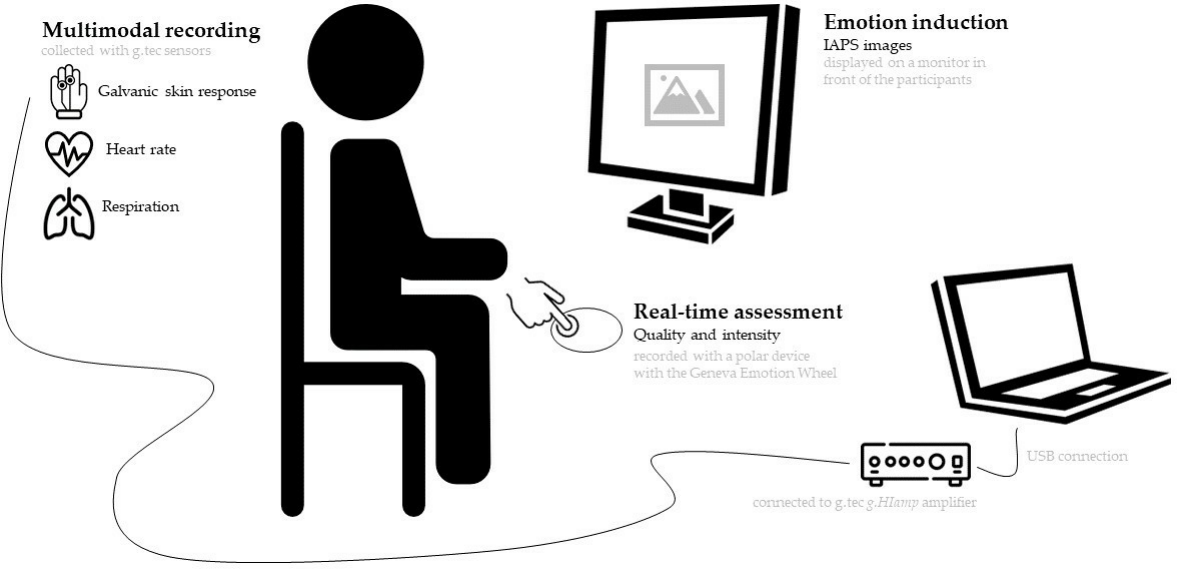
Schematic representation of the setup for data collection. Different biosensors are placed to collect GSR, HR and RSP. Emotions are elicited with images from the IAPS database, exhibited in a monitor in front of the participant. The subjective feeling is assessed with a polar feedback device based on the Geneva Emotion Wheel.

A schematic representation of the experimental setup can be found in Fig 3.

### Data processing

#### Pre-processing

As recommended by the manufacturer, we applied a low-pass filter of 30 Hz and a notch filter of 50 Hz to the signals from the sensors that measure GSR and pulse, and a band-pass filter with cut-off frequencies of 0.1 Hz and 30 Hz and a notch filter of 50 Hz to the respiration signals.

In the analysis, we excluded the first 2 seconds of each trial to account for the time participants took to adjust the quality at the beginning of the trials.

#### Feature extraction

We were interested in features able to give us information over time. Therefore, we contemplated the filtered GSR, the GSR derivative, the GSR running rate, the filtered HR, the HR derivative, the HR running rate, the RSP rate, the RSP rate derivative, the RSP running rate, inspiration time, expiration time, inhalation depth, and exhalation depth (see Fig 1(b)). The running rate is computed with respect to a reference interval that is moving along with the evaluation window as time proceeds. Besides the filtered GSR and HR and the derivatives, these features were extracted with the g.tec toolbox *g.HIsys*. Due to the quality of the RSP signal, inspiration time could not be obtained for 6 participants.

After the feature extraction step, we proceeded to a downsampling by a factor of 100 (resulting frequency of 2.56 Hz). In this way, we were able to attribute a physiological meaning to our results as discussed later.

#### Feature selection

We employed a subject-dependent sequential forward selection to select the most relevant features [31]. This was performed based on the quality of the results of the intensity prediction (more details below). In each iteration, we assessed which additional feature optimised the most the prediction of emotion intensity. As a cost function, we used the Pearson’s correlation coefficient; more specifically, we added features while the correlation would increase at least by 0.01. For computational reasons (computation time, more specifically), we combined a maximum of 3 features for each participant. In short, with just 1 feature, we tested all the features individually, but subsequently, we just combined the one obtaining the “best” result with the remaining features, and so on.

#### Intensity prediction

The emotion intensity for each individual quality was predicted with NARX models, in which the exogenous variables were combinations of the features described in Feature extraction. To train these models, the subjectively felt intensity assessed in real-time was used as ground-truth. Considering the nomenclature of Multimodal approach, intensity is *y* and the different physiological features are given by *u*. The regressors related to the intensity *y* were solely linear, while the ones for the physiological signals *u* were linear and quadratic. When combining more than one physiological feature, each exogenous variable *u*_*m*_ (with *m* ∈ {1, 2, 3}) had their own amount of regressors, based on their individual parameters $n_{u_{m}}$ and $n_{k_{m}}$.

To find the best fit, we trained NARX models with different combinations of parameters related to the number of regressors of each signal and to the delays of *u*_*m*_ in the reference to *y*. In other words, we assessed different combinations of the values that *n*_*y*_, $n_{u_{m}}$, and $n_{u_{k_{m}}}$ can take in (4). We allowed these parameters to take values between 1 and 11, which corresponds to time intervals between 0.39 s and 4.29 s (*f* = 2.56 Hz). In this sense, we considered short and long contributions from the already felt emotion intensity (*n*_*y*_) and from physiological outputs (*n*_*u*_). Moreover, we could assess whether those physiological changes (*n*_*u*_) resulted from fast or slow responses (*n*_*k*_).

All models were tested with a leave-one-out cross-validation (LOOCV) [31]. For each emotion quality, we had 7 available trials with varying intensity. Thus, for 7 times (all trials made once part of the test set), we used 6 trials as the training set and the remaining trial as test set. This process was repeated for the 21 subjects individually.

As mentioned in Feature selection, different numbers of features were considered. The attainment of the optimal parameters was dependent on this number, as we will explain next.

#### Parameter optimisation

In our previous work [27], we analysed each feature individually, obtaining the optimal parameters across subjects. Here, we could not simply combine the best combination of parameters for the individual features, since the different physiological features interact among them. Also, we used different methodologies to find the optimal parameters depending on the number of features and corresponding computational cost. As mentioned earlier, we did this by optimising the correlation between the intensity assessed by the participants and the intensity predicted by the model.

When considering just 1 feature, we only had to optimise 3 parameters (*n*_*y*_, $n_{u_{1}}$, and $n_{k_{1}}$) that could take integer values from 1 to 11 for each quality and individual, as well as for each physiological feature. We used an exhaustive search, since this task was still relatively fast to compute. However, when considering 2 and 3 features, we had to optimise 5 (adding $n_{u_{2}}$ and $n_{k_{2}}$) and 7 (adding $n_{u_{3}}$ and $n_{k_{3}}$) parameters, respectively, and an exhaustive search was no longer possible. Therefore, we used a genetic algorithm (GA) [31] to find the optimal parameters for each participant.

We used the GA to optimise the results obtained with the LOOCV mentioned in Intensity prediction of this subsection. This means that, for each subject, we computed the results 7 times (each time using 6 out of the 7 trials as a training set, and the remaining one as a test set) and then averaged these 7 values, aiming for the set of parameters that maximised this average. The maximum number of generations, *i.e*., the maximum number of iterations performed by the GA, was 1000 and 1400 for 2 and 3 features, respectively (200×number of parameters). This means that, if the algorithm does not find the best solution during the evolution process, it stops and provides the last generation solution and the best one. The process also stopped if the average cumulative change in value of the fitness function over 50 generations was less than 1 × 10^−6^, and if the constraint violation was less than 1 × 10^−3^.

For a better understanding, Fig 1(c) contains the pseudocode of our methodology and Fig 1(d) a representation of the LOOCV and respective outcome.

### Evaluation

Here, we are going to describe the methodology we used to obtain the results presented in the next section. Thus, for each method presented, there is its analog in Results.

#### Performance measures

We analysed the performance of our obtained models with the help of the correlation coefficient *r* computed between the predicted output and the ground-truth. With this, we obtained a measure of how close the predictions are in shape to the ground-truth. More specifically, we maximised the mean correlation across the iterations of the LOOCV: for a certain combination of parameters (*n*_*y*_, $n_{u_{1}}$, $n_{k_{1}}$, …, $n_{u_{m}}$, $n_{k_{m}}$), we averaged the 7 correlations resulting from the LOOCV.

In this work, we compared the results of the dynamic NARX model with the results of a classic sliding-window linear regression (LR). To make the LR reflect the dynamic behaviour and be comparable with the NARX models, we imposed the length of each window to be of 13 time points (which corresponds to approximately 5 s) and the step of 3 time points (approximately 1 s). In each time window, we considered the maximum, the minimum, the mean and the standard deviation as inputs of the model, and the mean intensity over the window as the output. Equivalently to the NARX models, we used a LOOCV to train the models and we considered the correlation *r* between the predicted output of the sliding-window LR and the ground-truth as a measure to assess performance. It is worth mentioning that, by using a larger window (*e.g*., 30 s), the performance of the LR model would improve, since we would be reducing the amount of noise; nonetheless, the dynamic effect would also be lost and the comparison with the dynamic model would not be adequate. The subject-dependent feature selection for this method was done similarly to the NARX models, adding physiological features based on their performance (mean correlation across LOOCV rotations). In both approaches, a physiological feature would only be kept if its addition improved the correlation in, at least, 0.01.

Finally, we compared the correlations obtained with the two methods with a Wilcoxon signed-rank test.

#### Feature ranking

Following the subject-dependent analysis, we examined which physiological features were more important to predict emotion intensity across subjects. To do so, for each physiological feature mentioned in Feature extraction, we calculated their occurrence among the optimal features across subjects for each emotion quality. Since this depends on the correlation obtained with each prediction method, this step was performed for NARX models and sliding-window LR separately.

With this, it was possible to obtain a ranking of the most important features in predicting intensity for each emotion quality using the two different prediction methods.

#### Intersubject analysis

After obtaining the feature ranking described above, we picked the most meaningful features (up to a maximum of 3 features) according to the respective prediction modality for each emotion quality, and conducted an intersubject analysis. To be considered meaningful, a feature needed to be present among the optimal features in the respective models for, at least, a quarter of the participants—which corresponds to 6 participants. With this analysis, we aimed not just to compare the effectiveness of both prediction models, but also to understand the feasibility of a dynamic intersubject model.

For this, we selected ∼ 70% of participants to be part of the training set, which corresponded to 14 participants and a total of 98 trials. For the test set, we used the remaining 7 participants and a total of 49 trials. We trained and tested the models exactly as for the intrasubject analysis, which means that we used a genetic algorithm to find the optimal *n*_*y*_, *n*_*u*_ and *n*_*k*_ for the prediction with the NARX model. In this case, we aimed to maximise the correlation across the 49 test trials. Hereby, *u*_1_, *u*_2_ and *u*_3_ represent the most meaningful features across subjects. For the sliding-window LR, the parameters were the same as for the intrasubject analysis (window of 13 time points and step of 3 time points). We repeated this process for 10 times, with random selection of participants for the training and the test datasets.

Finally, we compared the results of the 10 iterations of the two models using a Wilcoxon signed-rank test.

Please note that the most meaningful features are not necessarily the same for the NARX models and the sliding-window LR and, therefore, different sets of features were used to train the two different types of models.

#### Common combinations of features

Finally, we also analysed which features often occur together. To do so, we applied the *Apriori algorithm*, an algorithm used in frequent itemset mining [32]. Frequent itemset mining is a method of data analysis that was originally used to identify sets of products that are often bought together. Here, we aimed to identify which sets of features often appear together as the most relevant features to predict intensity. The Apriori algorithm has its name for following the Apriori property that, if an itemset is infrequent, all its supersets will be infrequent. A superset is a set that includes another set or sets. Considering *k* to be the number of items (with *k* ≥ 2), at the *k*^*th*^ iteration, the algorithm looks at the frequent itemsets with *k* items based on the frequent *k-1*-itemsets and determines whether they are still frequent [33]. All the infrequent itemsets are eliminated (pruning). In this work, we defined that for a set of features to be considered frequent, it would need to be present in, at least, one third of the participants (6 subjects). Also, since we combined up to a maximum of 3 physiological features, *k* could just go up to 3. This analysis was conducted separately for the 3 different qualities and for the 2 types of modelling approaches (NARX models and sliding-window LR).

## Results

### Performance measures

Tables 2–4 show the comparison of the two multimodal subject-dependent approaches (NARX models and sliding-window LR) for emotion recognition for qualities 1, 2 and 3. For each subject, we give an overview of the features that reached the best performance and corresponding achieved correlation, using nonlinear autoregressive exogenous models (left) and a sliding-window linear regression (right). For the NARX models, the optimal number of regressors of the intensity *y* and of the physiological features *u*_*m*_, as well as the delay of *u*_*m*_ with respect to *y*, are presented in these tables that reflect the results for each individual. Please note that, for each subject (*e.g*., Participant001), the parameters and correlation in the first row correspond to the first optimal feature (Filtered HR, in this case). In the following rows, they correspond to the addition of the new optimal feature to the previous ones (2^nd^ row: results with Filtered HR and RSP running rate; 3^rd^ row: results with Filtered HR, RSP running rate and Inhalation depth). The same applies to the results obtained with the sliding-window LR.

**Table 2:** Summary of the results of the individual models obtained for each participant for Happiness/Joy (Q1): Optimal features, corresponding parameters and obtained correlation coefficients for NARX models, and optimal features and obtained correlation coefficients for sliding-window linear regression. For each participant, each row corresponds to the results of the respective feature in addition to the previous ones.

Participants	NARX models									Linear regression	
	Optimal features	Parameters							Correlation	Optimal features	Correlation
		*n*_*y*_	$n_{u_{1}}$	$n_{k_{1}}$	$n_{u_{2}}$	$n_{k_{2}}$	$n_{u_{3}}$	$n_{k_{3}}$			
001	Filtered HR + RSP running rate + Inhalation depth	11 11 11	10 10 10	11 11 11	– 8 7	– 2 2	– – 11	– – 11	0.431 0.530 0.563	RSP running rate + HR derivative + HR running rate	0.194 0.218 0.234
002	Filtered HR + GSR running rate + RSP derivative	11 9 10	11 11 11	9 9 9	– 1 1	– 11 11	– – 5	– – 6	0.515 0.568 0.591	Filtered GSR	0.256
003	Filtered HR	10	1	9	–	–	–	–	0.757	GSR running rate + Exhalation depth + Expiration time + GSR derivative + RSP running rate	0.108 0.194 0.272 0.379 0.443
004	Filtered HR + RSP derivative + HR running rate	9 7 8	10 11 10	10 11 10	– 10 7	– 5 11	– – 2	– – 2	0.635 0.646 0.668	GSR running rate + HR running rate + HR derivative + Inhalation depth	0.155 0.274 0.306 0.371
005	Expiration time + GSR derivative + Filtered HR	6 11 2	10 6 5	11 6 5	– 10 11	– 7 9	– – 2	– – 10	0.575 0.639 0.666	Filtered GSR + Exhalation depth + GSR derivative + Expiration time	0.214 0.267 0.305 0.354
006	Filtered HR + GSR derivative	11 1	10 11	11 10	– 3	– 2	– –	– –	0.789 0.835	Exhalation depth	0.230
007	RSP rate + Filtered HR + GSR running rate	10 4 5	3 10 9	7 11 10	– 9 8	– 3 3	– – 2	– – 5	0.459 0.523 0.544	RSP derivative + GSR running rate	0.149 0.181
008	RSP running rate	1	11	1	–	–	–	–	0.623	Filtered HR + Exhalation depth + Expiration time + RSP rate	0.190 0.334 0.374 0.388
009	RSP running rate + Inhalation depth	1 3	10 6	2 3	– 6	– 3	– –	– –	0.504 0.539	Filtered HR	0.149
010	Filtered HR + HR derivative	1 1	3 9	9 10	– 3	– 2	– –	– –	0.557 0.616	GSR running rate + RSP derivative + RSP running rate + HR derivative	0.132 0.159 0.217 0.248
011	Inhalation depth + RSP derivative	2 5	8 7	10 11	– 2	– 8	– –	– –	0.653 0.697	HR running rate + Exhalation depth	0.161 0.251
012	Inhalation depth + GSR running rate + Filtered HR	1 10 1	8 2 1	11 8 11	– 3 4	– 11 2	– – 11	– – 11	0.806 0.850 0.866	GSR running rate + Expiration time + RSP running rate + Filtered GSR + GSR derivative	0.351 0.379 0.406 0.522 0.709
013	Filtered GSR + Filtered HR + Expiration time	3 9 3	5 10 8	4 4 1	– 9 4	– 11 10	– – 9	– – 8	0.506 0.586 0.703	GSR running rate + HR derivative	0.303 0.358
014	Filtered HR + RSP derivative + Exhalation depth	1 8 8	11 11 10	1 9 11	– 11 5	– 1 1	– – 11	– – 10	0.607 0.694 0.719	GSR running rate + HR derivative	0.198 0.208
015	Inspiration time + GSR running rate	1 4	3 3	7 8	– 2	– 7	– –	– –	0.631 0.645	GSR derivative + Inspiration time	0.160 0.171
016	RSP rate + Inhalation depth + RSP running rate	1 10 7	5 11 11	1 9 9	– 11 11	– 5 5	– – 6	– – 5	0.575 0.665 0.726	GSR running rate + HR derivative	0.218 0.346
017	Inspiration time + GSR derivative + Expiration time	8 6 6	3 1 1	10 9 3	– 3 1	– 10 3	– – 2	– – 10	0.501 0.568 0.622	Filtered GSR + Filtered HR + HR running rate	0.232 0.367 0.402
018	Filtered HR + Expiration time + RSP running rate	1 6 5	7 10 9	1 10 10	– 10 10	– 1 1	– – 5	– – 11	0.506 0.598 0.690	Inspiration time + HR derivative + RSP derivative	0.170 0.214 0.229
019	Filtered HR + GSR derivative + Exhalation depth	10 2 2	2 3 8	5 9 6	– 8 3	– 9 7	– – 7	– – 9	0.513 0.630 0.662	RSP derivative	0.128
020	Expiration time + Inspiration time	1 2	1 4	1 1	– 7	– 11	– –	– –	0.475 0.632	Inspiration time + RSP derivative	0.097 0.138
021	Filtered HR + HR running rate + RSP rate	10 11 11	11 10 10	10 11 11	– 10 8	– 1 1	– – 2	– – 8	0.542 0.571 0.593	RSP rate + RSP derivative	0.027 0.039

**Table 3:** Summary of the results of the individual models obtained for each participant for Disappointment/Regret (Q2): Optimal features, corresponding parameters and obtained correlation coefficients for NARX models, and optimal features and obtained correlation coefficients for sliding-window linear regression. For each participant, each row corresponds to the results of the respective feature in addition to the previous ones.

Participants	NARX models									Linear regression	
	Optimal features	Parameters							Correlation	Optimal features	Correlation
		*n*_*y*_	$n_{u_{1}}$	$n_{k_{1}}$	$n_{u_{2}}$	$n_{k_{2}}$	$n_{u_{3}}$	$n_{k_{3}}$			
001	Filtered GSR + RSP running rate + HR derivative	9 3 10	3 1 11	5 1 5	– 11 9	– 2 4	– – 10	– – 10	0.598 0.634 0.717	Inspiration time + Expiration time	0.229 0.295
002	Expiration time + GSR running rate + RSP rate	3 11 2	9 2 9	11 11 11	– 3 8	– 10 6	– – 8	– – 3	0.408 0.488 0.516	Filtered HR	0.0530
003	Filtered HR + RSP running rate + RSP derivative	10 2 7	2 4 3	7 11 5	– 10 1	– 8 11	– – 5	– – 7	0.713 0.739 0.754	GSR running rate + Filtered GSR + Inhalation depth + Filtered HR	0.107 0.164 0.236 0.273
004	Filtered HR + Expiration time + HR running rate	10 3 5	2 8 10	9 9 10	– 9 5	– 2 5	– – 1	– – 4	0.567 0.640 0.726	Filtered GSR + GSR derivative	0.364 0.432
005	Expiration time + HR running rate	3 3	9 11	9 7	– 1	– 7	– –	– –	0.684 0.698	RSP derivative + Expiration time	0.158 0.189
006	Expiration time + Filtered GSR	9 8	11 11	8 11	– 10	– 8	– –	– –	0.761 0.786	Filtered GSR + HR derivative	0.215 0.267
007	RSP rate + RSP derivative	5 11	2 6	9 11	– 4	– 5	– –	– –	0.720 0.748	RSP derivative + RSP running rate + RSP rate + HR derivative	0.105 0.167 0.241 0.270
008	Filtered GSR + GSR derivative	3 3	3 9	10 11	– 8	– 10	– –	– –	0.565 0.731	GSR running rate + GSR derivative	0.0983 0.136
009	Expiration time + HR running rate + GSR running rate	7 3 4	3 10 9	6 1 1	– 11 11	– 11 10	– – 3	– – 3	0.484 0.664 0.717	Expiration time + HR derivative + RSP rate + Filtered HR + HR running rate	0.102 0.132 0.184 0.228 0.262
010	Filtered GSR + Inspiration time + HR running rate	2 3 5	10 8 10	11 10 11	– 3 11	– 4 2	– – 9	– – 8	0.521 0.601 0.670	GSR running rate + Filtered GSR + Exhalation depth	0.155 0.234 0.264
011	Expiration time + Filtered GSR + Exhalation depth	3 2 3	11 7 9	7 11 9	– 4 2	– 10 8	– – 1	– – 3	0.533 0.603 0.628	HR derivative + Expiration time	0.0911 0.132
012	Expiration time	10	11	11	–	–	–	–	0.651	RSP derivative + RSP rate + HR running rate + Filtered GSR + Expiration time	0.0998 0.152 0.186 0.233 0.387
013	Expiration time + GSR running rate + RSP rate	5 5 2	10 11 11	10 9 8	– 1 2	– 2 2	– – 10	– – 1	0.540 0.603 0.629	Filtered GSR + Exhalation depth + RSP running rate + Expiration time	0.246 0.302 0.329 0.359
014	Inspiration time + HR derivative + Exhalation depth	11 5 1	11 1 11	11 7 11	– 6 5	– 7 5	– – 6	– – 7	0.609 0.635 0.696	GSR running rate + Expiration time + HR derivative + Filtered HR	0.0619 0.149 0.191 0.222
015	Filtered GSR + GSR derivative + GSR running rate	1 1 3	5 10 10	5 11 11	– 6 3	– 5 6	– – 8	– – 5	0.577 0.689 0.716	Inhalation depth + RSP rate	0.101 0.136
016	GSR running rate + Filtered HR + Expiration time	1 6 2	10 1 3	9 10 4	– 1 11	– 2 10	– – 6	– – 1	0.491 0.584 0.634	Filtered GSR + Expiration time	0.437 0.528
017	Inspiration time + GSR derivative + Filtered HR	7 3 2	2 5 1	9 9 8	– 8 10	– 9 8	– – 3	– – 9	0.628 0.706 0.760	Expiration time + HR running rate	0.123 0.149
018	Filtered HR + Expiration time + Inspiration time	8 3 5	11 11 10	11 8 10	– 9 10	– 1 1	– – 6	– – 10	0.559 0.699 0.759	Filtered HR	0.172
019	Expiration time + Inspiration time + RSP running rate	7 7 1	2 11 11	8 11 5	– 4 2	– 4 8	– – 11	– – 11	0.654 0.747 0.761	Inspiration time + HR running rate	0.203 0.283
020	Exhalation depth + Filtered GSR + RSP running rate	7 7 11	3 1 5	6 4 11	– 1 1	– 9 3	– – 11	– – 10	0.513 0.562 0.583	Filtered HR	0.096
021	Inhalation depth	9	2	8	–	–	–	–	0.477	GSR running rate + Inspiration time + Exhalation depth + HR running rate	0.320 0.382 0.412 0.459

**Table 4:** Summary of the results of the individual models obtained for each participant for Worry/Fear (Q3): Optimal features, corresponding parameters and obtained correlation coefficients for NARX models, and optimal features and obtained correlation coefficients for sliding-window linear regression. For each participant, each row corresponds to the results of the respective feature in addition to the previous ones.

Participants	NARX models									Linear regression	
	Optimal features	Parameters							Correlation	Optimal features	Correlation
		*n*_*y*_	$n_{u_{1}}$	$n_{k_{1}}$	$n_{u_{2}}$	$n_{k_{2}}$	$n_{u_{3}}$	$n_{k_{3}}$			
001	Filtered GSR + Exhalation depth + GSR derivative	10 9 5	3 3 1	8 8 11	– 1 4	– 5 10	– – 11	– – 7	0.465 0.522 0.611	GSR derivative + Inspiration time + Expiration time + Inhalation depth	0.0367 0.0786 0.108 0.127
002	Exhalation depth + Filtered HR + GSR running rate	3 10 9	7 4 1	11 2 2	– 11 11	– 9 9	– – 7	– – 1	0.382 0.457 0.492	Filtered HR + GSR derivative + RSP rate	0.0875 0.130 0.178
003	Inspiration time + HR derivative + Inhalation depth	2 1 5	8 11 9	9 10 10	– 3 2	– 1 2	– – 6	– – 6	0.511 0.584 0.616	RSP derivative + HR derivative	0.269 0.307
004	Filtered GSR + GSR running rate + Filtered HR	7 11 6	6 10 7	5 4 6	– 3 5	– 3 1	– – 9	– – 11	0.518 0.562 0.595	Filtered GSR + GSR derivative + Exhalation depth + Expiration time	0.258 0.276 0.385 0.402
005	Expiration time + Inhalation depth + Filtered HR	8 3 5	11 9 11	8 10 10	– 8 8	– 2 2	– – 1	– – 10	0.511 0.639 0.669	Filtered GSR + HR running rate	0.108 0.169
006	Filtered HR + RSP rate	11 10	6 11	11 11	– 5	– 11	– –	– –	0.778 0.823	Inspiration time + RSP running rate	0.382 0.405
007	Filtered HR + RSP rate + RSP derivative	3 6 11	11 11 11	0 11 11	– 8 5	– 3 5	– – 11	– – 3	0.493 0.557 0.589	Filtered GSR	0.420
008	Expiration time + RSP rate + Inspiration time	2 6 7	11 11 11	9 11 11	– 11 9	– 1 2	– – 6	– – 2	0.576 0.672 0.703	GSR running rate + Filtered HR + Filtered GSR + GSR derivative	0.127 0.187 0.217 0.242
009	RSP running rate + Expiration time + Filtered GSR	8 4 2	4 8 10	10 11 8	– 1 1	– 9 10	– – 2	– – 10	0.454 0.572 0.628	RSP rate + Expiration time	0.127 0.173
010	Filtered HR + RSP running rate + Inspiration time	8 8 8	8 8 4	9 10 1	– 8 7	– 10 10	– – 9	– – 9	0.612 0.636 0.665	Inhalation depth	0.182
011	RSP rate + GSR derivative + RSP running rate	10 2 2	4 2 3	9 10 11	– 6 1	– 10 11	– – 2	– – 2	0.459 0.500 0.511	GSR running rate + Inspiration time + HR derivative	0.234 0.311 0.337
012	RSP running rate	11	4	6	–	–	–	–	0.709	GSR running rate + Filtered HR + Filtered GSR + Expiration time	0.174 0.198 0.224 0.252
013	Inhalation depth + GSR running rate + RSP rate	3 4 10	5 5 3	5 5 4	– 11 10	– 10 10	– – 5	– – 9	0.532 0.606 0.651	Filtered HR + GSR running rate + HR derivative	0.264 0.276 0.294
014	Filtered GSR + GSR derivative + Inhalation depth	1 2 2	1 11 9	1 11 11	– 11 10	– 10 8	– – 8	– – 11	0.628 0.716 0.758	HR running rate + GSR derivative + Inhalation depth + Filtered GSR	0.206 0.274 0.392 0.450
015	Inspiration time + Filtered GSR + GSR running rate	10 10 7	2 6 9	8 10 6	– 7 9	– 4 7	– – 6	– – 11	0.470 0.538 0.575	Filtered GSR	0.262
016	Exhalation depth + Expiration time + Inhalation depth	6 9 8	4 4 4	1 3 5	– 11 10	– 10 11	– – 3	– – 4	0.525 0.560 0.575	RSP derivative + GSR derivative	0.102 0.133
017	Inspiration time + Filtered HR + RSP running rate	9 6 4	3 5 4	10 3 6	– 10 11	– 11 11	– – 11	– – 2	0.473 0.585 0.619	HR running rate + Inhalation depth + Filtered GSR	0.016 0.077 0.261
018	GSR running rate + Inspiration time + Exhalation depth	10 4 6	5 8 11	9 9 6	– 2 2	– 10 10	– – 1	– – 11	0.542 0.589 0.609	HR derivative	0.341
019	RSP rate + HR running rate + Exhalation depth	9 9 9	8 7 8	8 9 5	– 1 11	– 6 11	– – 6	– – 10	0.467 0.490 0.537	RSP derivative + Expiration time	0.182 0.204
020	Inhalation depth + Inspiration time + Expiration time	4 1 1	6 11 2	9 11 1	– 11 9	– 3 11	– – 8	– – 3	0.569 0.652 0.737	Filtered HR + Filtered GSR	0.304 0.332
021	Filtered HR + Exhalation depth + Expiration time	7 11 7	8 11 4	10 10 9	– 9 4	– 5 7	– – 2	– – 10	0.475 0.546 0.636	HR running rate + GSR derivative	0.142 0.166

As we mentioned, NARX models just accounted for a maximum of 3 features due to computational restrictions. On the other hand, the baseline approach was allowed to add features until reaching its maximum potential. Even benefitting this aspect in the sliding-window linear regression, NARX models outperformed their results for all subjects and qualities (*p*-value <0.001 for Q1, as well as for Q2 and Q3).

In the case of NARX models, it is possible to observe in Tables 2–4 that the correlations varied as follows: for Q1, from 0.539 (Participant009) to 0.866 (Participant012); for Q2, from 0.477 (Participant021) to 0.786 (Participant006); and, for Q3, from 0.492 (Participant002) to 0.823 (Participant007). For sliding-window LR results, they varied, respectively for Q1, Q2, and Q3, in the following way: from 0.039 (Participant021) to 0.709 (Participant012); from 0.0530 (Participant002) to 0.528 (Participant016); and from 0.127 (Participant001) to 0.450 (Participant014).

**Fig 4:**
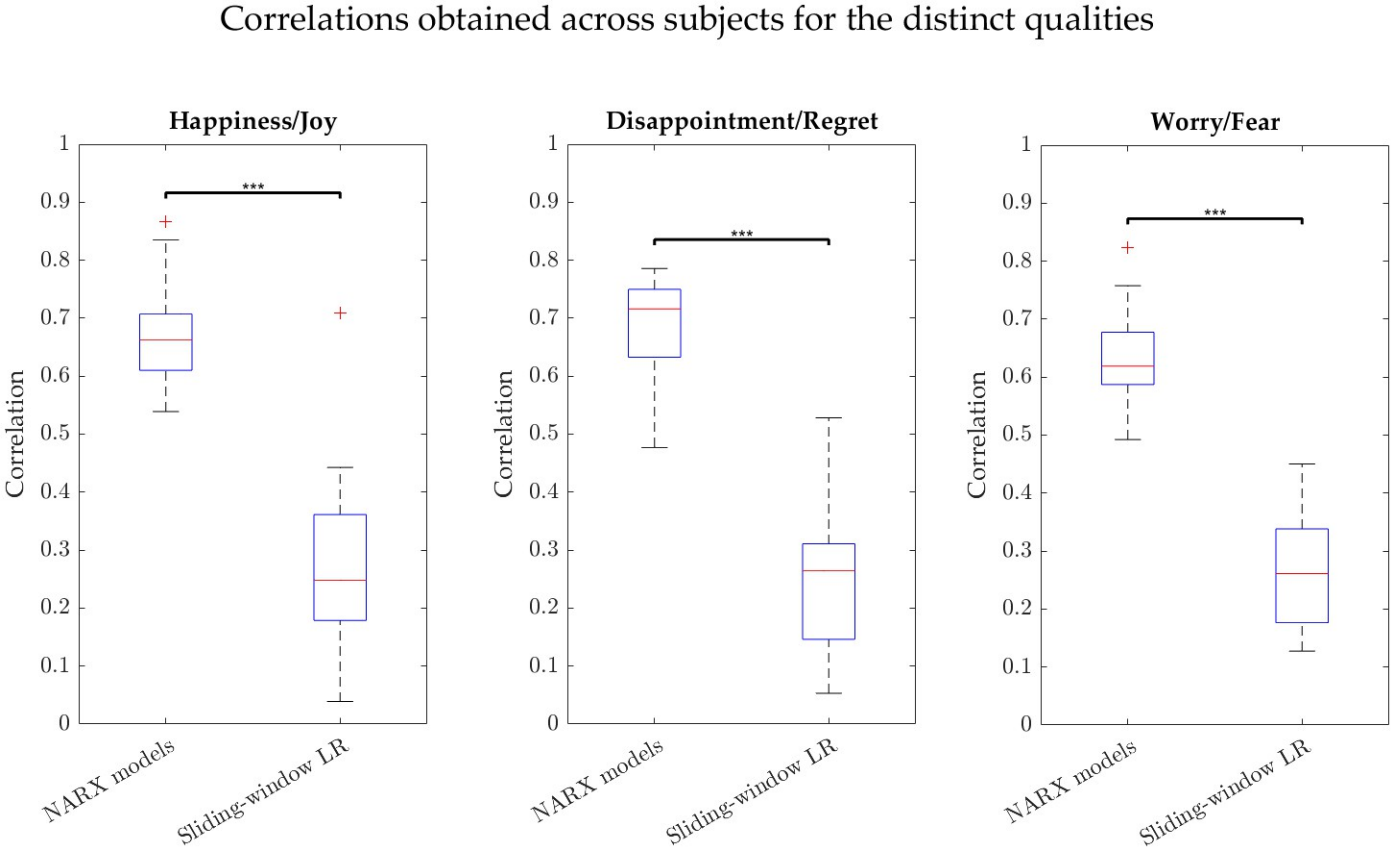
Boxplots of the best subject-dependent correlations in the intensity prediction. Results for both NARX models (left) and sliding-window linear regression (right) for Happiness/Joy (Q1), Disappointment/Regret (Q2), and Worry/Fear (Q3).

The medians (median absolute deviations) of the subject-dependent best correlations assumed the following values for NARX models: 0.662 (0.046), 0.716 (0.044) and 0.619 (0.044) for Q1, Q2 and Q3, respectively, and for sliding-window LR: 0.248 (0.099), 0.264 (0.095), 0.261 (0.080) for Q1, Q2 and Q3, respectively. The boxplots representing the best subject-dependent correlations can be found in Fig 4 for the different qualities.

### Feature ranking

**Fig 5:**
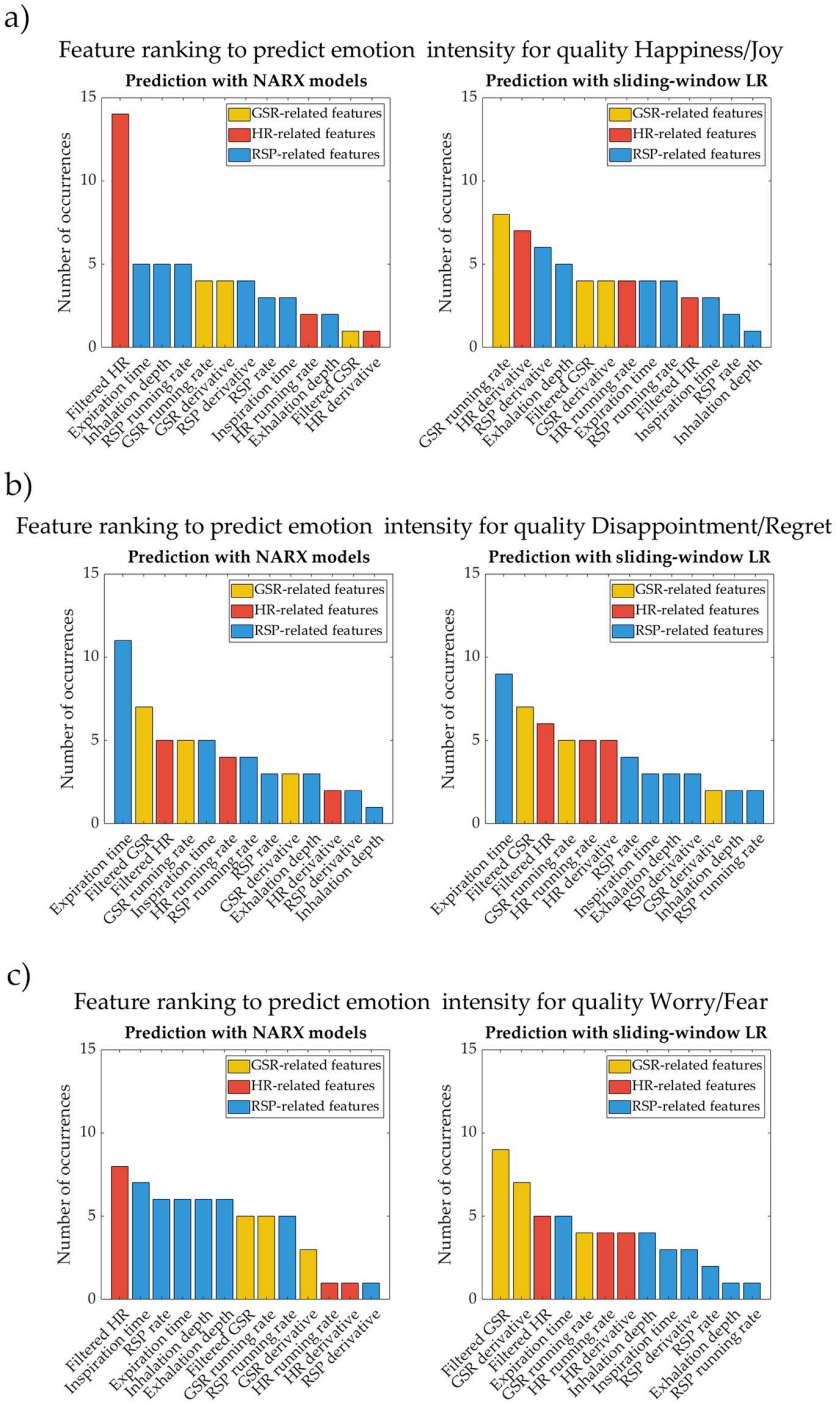
Ranking of the importance of the different physiological features in the prediction of emotion intensity. Results for both NARX models (left) and sliding-window linear regression (right) for a) Happiness/Joy (Q1), b) Disappointment/Regret (Q2), c) and Worry/Fear (Q3). Physiological features related to galvanic skin response are represented in yellow, to heart rate in red, and to respiration in blue.

From Tables 2–4, as well as the histogram shown in Fig 5, we can determine which physiological features were more relevant to predict emotion intensity for the different qualities and for each of the approaches (NARX models or sliding-window LR).

In the case of Q1 (Happiness/Joy), filtered heart rate was predominant (14 participants) for NARX models, and GSR running rate (8 participants), HR derivative (7 participants) and RSP derivative (6 participants) for the sliding-window LR.

Considering Q2 (Disappointment/Regret), expiration time (11 participants) and filtered GSR (7 participants) were the features giving more information for the prediction of intensity using NARX models, and expiration time (9 participants), filtered GSR (7 participants) and filtered HR (6 participants) for the sliding-window LR.

Finally, for Q3 (Worry/Fear), the features contributing more to the prediction of intensity with NARX models did not have any feature as “prominent” as for the previous qualities: filtered HR (8 participants), inspiration time (7 participants), RSP rate, expiration time, inhalation depth and exhalation depth (6 participants each). With the sliding-window LR, the most prominent features were filtered GSR (9 participants) and GSR derivative (7 participants).

### Intersubject analysis

Tables 5–7 show the results of the intersubject analysis for all the qualities, using the most relevant features resulting from above. In the case of the NARX and Q3, the 2nd most frequent feature was inspiration time—a feature that was not calculated for 6 participants, as mentioned in Data processing. For this reason, it was not included in the intersubject analysis. Moreover, there were 4 different physiological features in the 3rd place of the ranking (RSP rate, expiration time, inhalation depth and exhalation depth). Therefore, six different models were considered, in which the second and the third feature were combinations of those (see Table 7).

**Table 5:** Summary of the results (optimal parameters and resulting correlation coefficients) of the intersubject models for the different selections of training and test sets for Happiness/Joy (Q1), obtained with filtered HR (*u*_1_) for NARX models and with GSR running rate, HR derivative and RSP derivative for the sliding-window linear regression.

		NARX models (filtered HR)		Sliding-window LR (combination of GSR running rate, HR derivative and RSP derivative)
Training set (subjects’ indexes)	Test set (subjects’ indexes)	Parameters [*n*_*y*_,$n_{u_{1}}$,$n_{k_{1}}$]	Mean correlation	Mean correlation
001, 002, 003, 005, 006, 007, 008, 011, 014, 015, 016, 017, 019, 021	004, 009, 010, 012, 013, 018, 020	[7, 8, 11]	0.572	0.0218
001, 003, 004, 005, 006, 007, 010, 012, 014, 015, 017, 018, 020, 021	002, 008, 009, 011, 013, 016, 019	[8, 11, 9]	0.516	-0.0128
002, 003, 004, 005, 006, 007, 008, 009, 011, 014, 015, 016, 017, 019	001, 010, 012, 013, 018, 020, 021	[4, 7, 11]	0.592	-8.53e-3
001, 004, 005, 008, 009, 010, 011, 012, 013, 015, 016, 017, 019, 021	002, 003, 006, 007, 014, 018, 020	[3, 9, 8]	0.551	-6.57e-3
002, 003, 006, 008, 010, 012, 013, 014, 015, 016, 017, 18, 019, 020	001, 004, 005, 007, 009, 011, 021	[3, 9, 9]	0.516	-0.0147
001, 002, 004, 006, 009, 010, 012, 013, 014, 015, 016, 017, 018, 019	003, 005, 007, 008, 011, 020, 021	[11, 8, 11]	0.486	-0.0154
001, 002, 003, 004, 005, 007, 008, 010, 011, 012, 013, 014, 016, 021	006, 009, 015, 017, 018, 019, 020	[11, 9, 11]	0.579	0.0102
001, 002, 003, 007, 008, 009, 010, 011, 013, 014, 015, 017, 018, 021	004, 005, 006, 012, 016, 019, 020	[11, 11, 10]	0.596	-8.58e-3
001, 004, 005, 006, 009, 010, 012, 013, 016, 017, 018, 019, 020, 021	002, 003, 007, 008, 011, 014, 015	[3, 6, 11]	0.528	0.0212
001, 002, 003, 004, 008, 009, 010, 011, 015, 016, 018, 019, 020, 021	005, 006, 007, 012, 013, 014, 017	[9, 11, 10]	0.610	7.42e-3

**Table 6:** Summary of the results (optimal parameters and resulting correlation coefficients) of the intersubject models for the different selections of training and test sets for Disappointment/Regret (Q2), obtained with filtered GSR (*u*_1_) and expiration time (*u*_2_) for NARX models and with filtered GSR, filtered HR and expiration time for the sliding-window linear regression.

		NARX models (filtered GSR and expiration time)		Sliding-window LR (combination of filtered GSR, filtered HR and expiration time)
Training set (subjects’ indexes)	Test set (subjects’ indexes)	Parameters [*n*_*y*_,$n_{u_{1}}$,$n_{k_{1}}$, $n_{u_{2}}$,$n_{k_{2}}$]	Mean correlation	Mean correlation
001, 002, 003, 005, 006, 007, 008, 011, 014, 015, 016, 017, 019, 021	004, 009, 010, 012, 013, 018, 020	[2, 2, 3, 3, 8]	0.505	-0.0174
001, 002, 003, 004, 005, 008, 009, 010, 012, 014, 016, 018, 020, 021	006, 007, 011, 013, 015, 017, 019	[7, 10, 4, 8, 8]	0.470	-0.0244
001, 002, 004, 005, 006, 009, 011, 014, 015, 017, 018, 019, 020, 021	003, 007, 008, 010, 012, 013, 016	[8, 7, 5, 11, 5]	0.416	-0.0431
001, 002, 003, 004, 005, 006, 008, 010, 011, 012, 016, 018, 019, 021	007, 009, 013, 014, 015, 017, 020	[8, 5, 9, 10, 6]	0.501	-0.0162
001, 002, 003, 010, 011, 012, 013, 014, 015, 016, 018, 019, 020, 021	004, 005, 006, 007, 008, 009, 017	[3, 2, 10, 5, 8]	0.495	-7.95e-3
002, 003, 004, 005, 007, 008, 010, 011, 012, 016, 017, 018, 020, 021	001, 006, 009, 013, 014, 015, 019	[6, 4, 10, 8, 8]	0.541	0.0121
001, 002, 003, 004, 005, 007, 008, 009, 015, 016, 017, 019, 020, 021	006, 010, 011, 012, 013, 014, 018	[4, 6, 7, 11, 5]	0.448	-4.39e-3
001, 002, 003, 004, 005, 006, 007, 010, 012, 013, 015, 016, 018, 019	008, 009, 011, 014, 017, 020, 021	[10, 11, 5, 7, 6]	0.369	-2.28e-3
002, 003, 004, 005, 006, 007, 009, 010, 012, 014, 016, 018, 020, 021	001, 008, 011, 013, 015, 017, 019	[1, 11, 11, 2, 10]	0.627	0.0139
002, 003, 004, 006, 007, 008, 009, 010, 011, 013, 014, 019, 020, 021	001, 005, 012, 015, 016, 017, 018	[10, 9, 7, 9, 5]	0.491	-7.56e-3

**Table 7:** Summary of the results (optimal parameters and resulting correlation coefficients) of the intersubject models for the different selections of training and test sets for Worry/Fear (Q3). For NARX models, those were obtained with six different combinations of features: (i) filtered HR (*u*_1_), RSP rate (*u*_2_) and expiration time (*u*_3_), (ii) filtered HR (*u*_1_), RSP rate (*u*_2_) and inhalation depth (*u*_3_), (iii) filtered HR (*u*_1_), RSP rate (*u*_2_) and exhalation depth (*u*_3_), (iv) filtered HR (*u*_1_), expiration time (*u*_2_) and inhalation depth (*u*_3_), (v) filtered HR (*u*_1_), expiration time (*u*_2_) and exhalation depth (*u*_3_), and (vi) filtered HR (*u*_1_), inhalation depth (*u*_2_) and exhalation depth (*u*_3_). For the sliding-window linear regression, the combined features were filtered GSR and GSR derivative.

		NARX models (combination of filtered HR, RSP rate and expiration time)		NARX models (combination of filtered HR, RSP rate and inhalation depth)		NARX models (combination of filtered HR, RSP rate and exhalation depth)		NARX models (combination of filtered HR, expiration time and inhalation depth)		NARX models (combination of filtered HR, expiration time and exhalation depth)		NARX models (combination of filtered HR, inhalation depth and exhalation depth)		Sliding-window LR (combination of filtered GSR and GSR derivative)
Training set (subjects’ indexes)	Test set (subjects’ indexes)	Parameters [*n*_*y*_,$n_{u_{1}}$,$n_{k_{1}}$, $n_{u_{2}}$,$n_{k_{2}}$,$n_{u_{3}}$,$n_{k_{3}}$]	Mean correlation	Parameters [*n*_*y*_,$n_{u_{1}}$,$n_{k_{1}}$, $n_{u_{2}}$,$n_{k_{2}}$,$n_{u_{3}}$,$n_{k_{3}}$]	Mean correlation	Parameters [*n*_*y*_,$n_{u_{1}}$,$n_{k_{1}}$, $n_{u_{2}}$,$n_{k_{2}}$,$n_{u_{3}}$,$n_{k_{3}}$]	Mean correlation	Parameters [*n*_*y*_,$n_{u_{1}}$,$n_{k_{1}}$, $n_{u_{2}}$,$n_{k_{2}}$,$n_{u_{3}}$,$n_{k_{3}}$]	Mean correlation	Parameters [*n*_*y*_,$n_{u_{1}}$,$n_{k_{1}}$, $n_{u_{2}}$,$n_{k_{2}}$,$n_{u_{3}}$,$n_{k_{3}}$]	Mean correlation	Parameters [*n*_*y*_,$n_{u_{1}}$,$n_{k_{1}}$, $n_{u_{2}}$,$n_{k_{2}}$,$n_{u_{3}}$,$n_{k_{3}}$]	Mean correlation	Mean correlation
002, 005, 006, 007, 008, 009, 012, 014, 015, 016, 017, 019, 020, 021	001, 003, 004, 010, 011, 013, 018	[4, 10, 9, 1, 8, 6, 5]	0.620	[8, 9, 11, 4, 2, 9, 9]	0.610	[5, 11, 11, 2, 8, 4, 10]	0.625	[4, 10, 11, 4, 5, 6, 11]	0.608	[4, 11, 10, 7, 2, 2, 9]	0.602	[9, 8, 9, 5, 3, 1, 3]	0.606	-0.0174
001, 002, 003, 004, 005, 006, 007, 008, 010, 012, 014, 015, 016, 018	009, 011, 013, 017, 019, 020, 021	[8, 8, 10, 7, 11, 6, 4]	0.547	[7, 9, 8, 6, 8, 10, 8]	0.567	[8, 9, 9, 1, 10, 9, 10]	0.532	[9, 8, 9, 10, 2, 5, 6]	0.547	[8, 9, 10, 4, 7, 1, 1]	0.552	[11, 9, 8, 5, 1, 4, 7]	0.557	0.0184
001, 002, 003, 005, 007, 008, 009, 010, 014, 017, 018, 019, 020, 021	004, 006, 011, 012, 013, 015, 016	[4, 10, 11, 4, 5, 10, 2]	0.591	[7, 11, 11, 2, 4, 10, 1]	0.590	[10, 10, 10, 1, 5, 1, 3]	0.586	[8, 11, 11, 3, 3, 7, 2]	0.612	[4, 11, 11, 11, 6, 4, 9]	0.614	[8, 11, 11, 4, 3, 2, 7]	0.595	-7.05e-3
001, 002, 003, 004, 005, 009, 010, 012, 013, 015, 016, 017, 018, 019	006, 007, 008, 011, 014, 020, 021	[7, 11, 11, 1, 9, 11, 7]	0.645	[10, 9, 9, 2, 4, 3, 4]	0.622	[8, 9, 10, 2, 7, 4, 7]	0.630	[10, 9, 11, 6, 1, 3, 8]	0.634	[9, 9, 11, 10, 10, 5, 2]	0.634	[8, 11, 11, 1, 11, 7, 10]	0.642	6.24e-3
001, 002, 003, 004, 006, 007, 008, 012, 013, 014, 016, 018, 019, 020	005, 009, 010, 011, 015, 017, 021	[5, 9, 9, 9, 6, 6, 6]	0.583	[10, 9, 9, 10, 8, 5, 10]	0.572	[5, 10, 11, 8, 1, 9, 2]	0.571	[8, 9, 9, 5, 5, 3, 6]	0.582	[5, 10, 10, 9, 3, 4, 3]	0.568	[7, 11, 11, 5, 2, 2, 7]	0.562	-0.0175
001, 004, 005, 006, 007, 008, 009, 012, 013, 015, 016, 019, 020, 021	002, 003, 010, 011, 014, 017, 018	[10, 10, 10, 1, 7, 4, 3]	0.572	[9, 9, 10, 3, 2, 7, 4]	0.574	[8, 8, 11, 2, 6, 9, 9]	0.570	[9, 10, 10, 4, 5, 9, 10]	0.570	[9, 9, 11, 6, 3, 10, 9]	0.565	[10, 10, 11, 7, 9, 6, 2]	0.574	-6.69e-3
001, 002, 004, 006, 007, 008, 009, 010, 011, 013, 014, 018, 019, 020	003, 005, 012, 015, 016, 017, 021	[4, 11, 9, 9, 6, 8, 8]	0.542	[6, 10, 11, 2, 9, 3, 9]	0.548	[11, 8, 10, 2, 8, 8, 9]	0.549	[4, 10, 10, 8, 4, 3, 7]	0.578	[3, 11, 9, 9, 1, 11, 8]	0.584	[6, 7, 10, 7, 8, 6, 4]	0.565	4.06e-3
001, 004, 005, 006, 007, 008, 009, 010, 011, 013, 016, 018, 019, 021	002, 003, 012, 014, 015, 017, 020	[6, 11, 11, 5, 10, 11, 6]	0.609	[8, 11, 11, 1, 9, 5, 3]	0.613	[8, 11, 11, 1, 1, 5, 4]	0.617	[4, 11, 11, 10, 8, 11, 2]	0.606	[6, 10, 11, 10, 4, 3, 8]	0.614	[8, 11, 11, 3, 7, 4, 2]	0.616	0.0322
001, 002, 003, 004, 006, 007, 009, 013, 014, 015, 017, 019, 020, 021	005, 008, 010, 011, 012, 016, 018	[5, 10, 10, 1, 6, 9, 2]	0.607	[5, 10, 8, 5, 8, 10, 6]	0.575	[8, 9, 9, 8, 10, 1, 7]	0.580	[1, 10, 7, 10, 5, 1, 10]	0.630	[1, 8, 9, 9, 6, 4, 6]	0.603	[7, 9, 8, 10, 7, 3, 10]	0.586	0.0230
002, 005, 006, 007, 008, 009, 010, 011, 013, 014, 017, 018, 019, 021	001, 003, 004, 012, 015, 016, 020	[3, 11, 9, 10, 11, 11, 2]	0.598	[7, 9, 9, 2, 6, 3, 4]	0.589	[6, 11, 10, 2, 2, 3, 4]	0.588	[5, 10, 11, 8, 3, 7, 11]	0.598	[5, 11, 11, 11, 2, 9, 11]	0.599	[6, 8, 10, 6, 3, 7, 8]	0.578	-0.0194

As possible to see in Tables 5–7, the correlations of the sliding-window LR were very low (close to 0) for all qualities: from -0.0154 to 0.0218 in the case of Q1, from -0.0431 to 0.0139 in Q2, and from -0.0194 to 0.0322 in Q3. On the other hand, NARX models achieved the following range of correlations: from 0.486 to 0.610 in Q1, from 0.369 to 0.627 in Q2, and from 0.532 to 0.642 across all combinations of Q3. In sum, the dynamic approach showed significantly higher performance than the linear regression, for all qualities (*p*-values < 0.001 for Q1 and Q2, as well as for the six different combinations of features of Q3).

**Fig 6:**
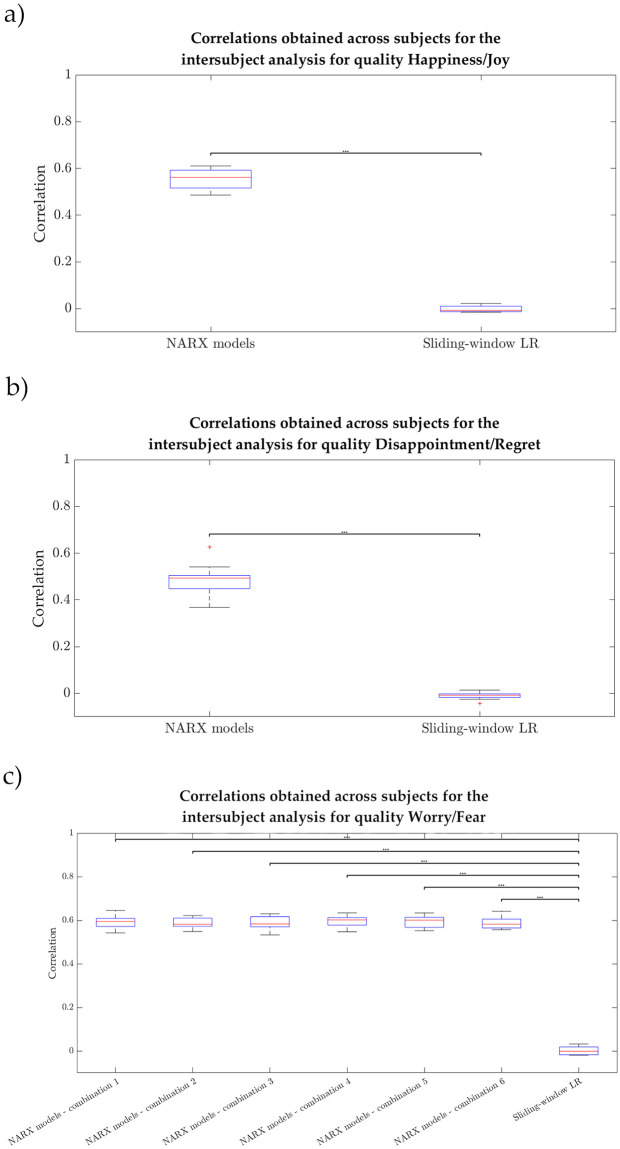
Boxplots of the correlations across iterations in the intersubject intensity prediction. a) For Happiness/Joy (Q1), NARX models use filtered HR (left), and sliding-window linear regression uses GSR running rate, HR derivative and RSP derivative (right). b) For Disappointment/Regret (Q2), NARX models use expiration time and filtered GSR (left), and sliding-window linear regression uses expiration time, filtered GSR and filtered HR (right). c) For Worry/Fear (Q3), sliding-window linear regression uses filtered GSR and GSR derivative (the rightmost group), and NARX models use different combinations, namely: combination 1 (filtered HR, RSP rate and expiration time), combination 2 (filtered HR, RSP rate and inhalation depth), combination 3 (filtered HR, RSP rate an exhalation depth), combination 4 (filtered HR, expiration time and inhalation depth), combination 5 (filtered HR, expiration time and exhalation depth) and combination 6 (filtered HR, inhalation depth and exhalation depth).

Not surprisingly, the medians (median absolute deviations) were as follows: for sliding-window LR, -7.55e-3 (7.40e-3), -7.76e-3 (9.08e-3) and -1.31e-3 (0.0161) for Q1, Q2 and Q3, respectively; for NARX models, 0.561 (0.0340) for Q1, 0.493 (0.0343) for Q2, and 0.595 (0.0184), 0.582 (0.0123), 0.583 (0.0235), 0.602 (0.0220), 0.600 (0.0152) and 0.582 (0.0185) for the different feature combinations of Q3. The respective boxplots can be found in Fig 6, where the described distributions can be observed.

**Fig 7:**
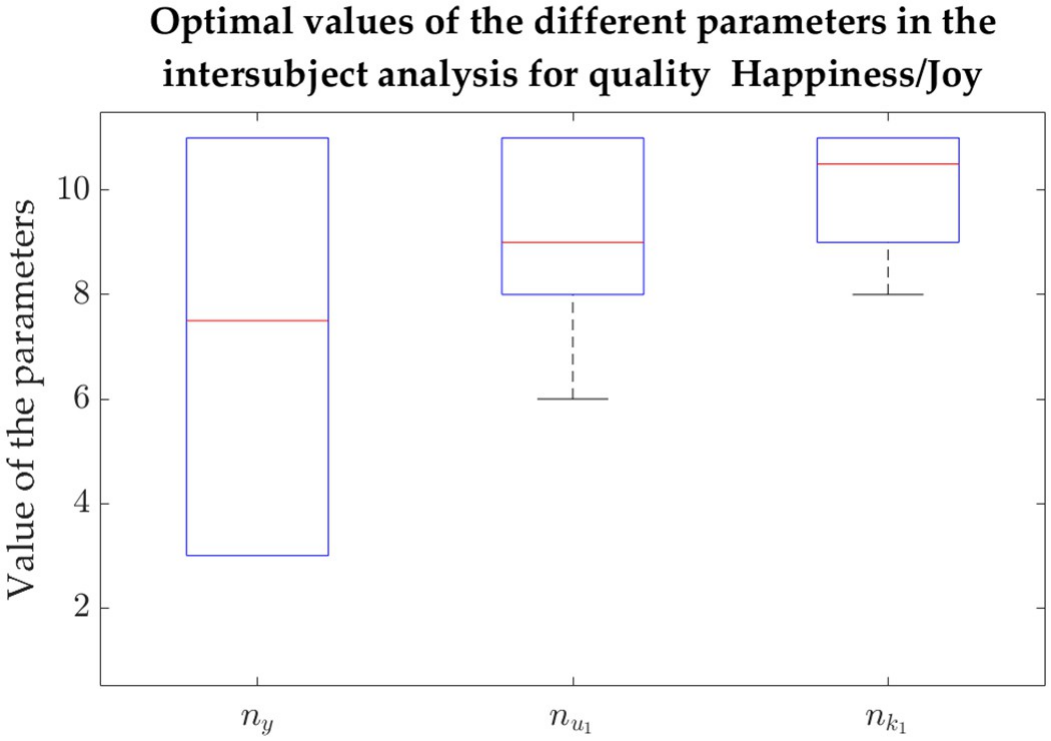
Boxplots of the values taken by the different parameters related to the intersubject models for Happiness/Joy (Q1). (a) The intensity prediction for Q1 considered regressors from emotion intensity *y* (*n*_*y*_) as well as from filtered HR ($n_{u_{1}}$). The delays between this physiological feature and the emotion intensity are given by $n_{k_{1}}$.

**Fig 8:**
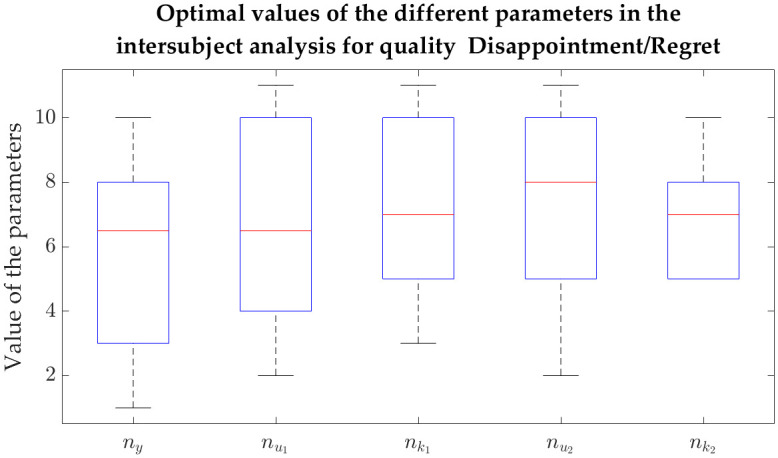
Boxplots of the values taken by the different parameters related to the intersubject models for Disappointment/Regret (Q2). The intensity prediction for Q2 considered regressors from emotion intensity *y* (*n*_*y*_) as well as from filtered GSR ($n_{u_{1}}$) and expiration time ($n_{u_{2}}$). The delays between each one of these physiological features and the emotion intensity are given by the respective $n_{k_{1}}$ and $n_{k_{2}}$.

**Fig 9:**
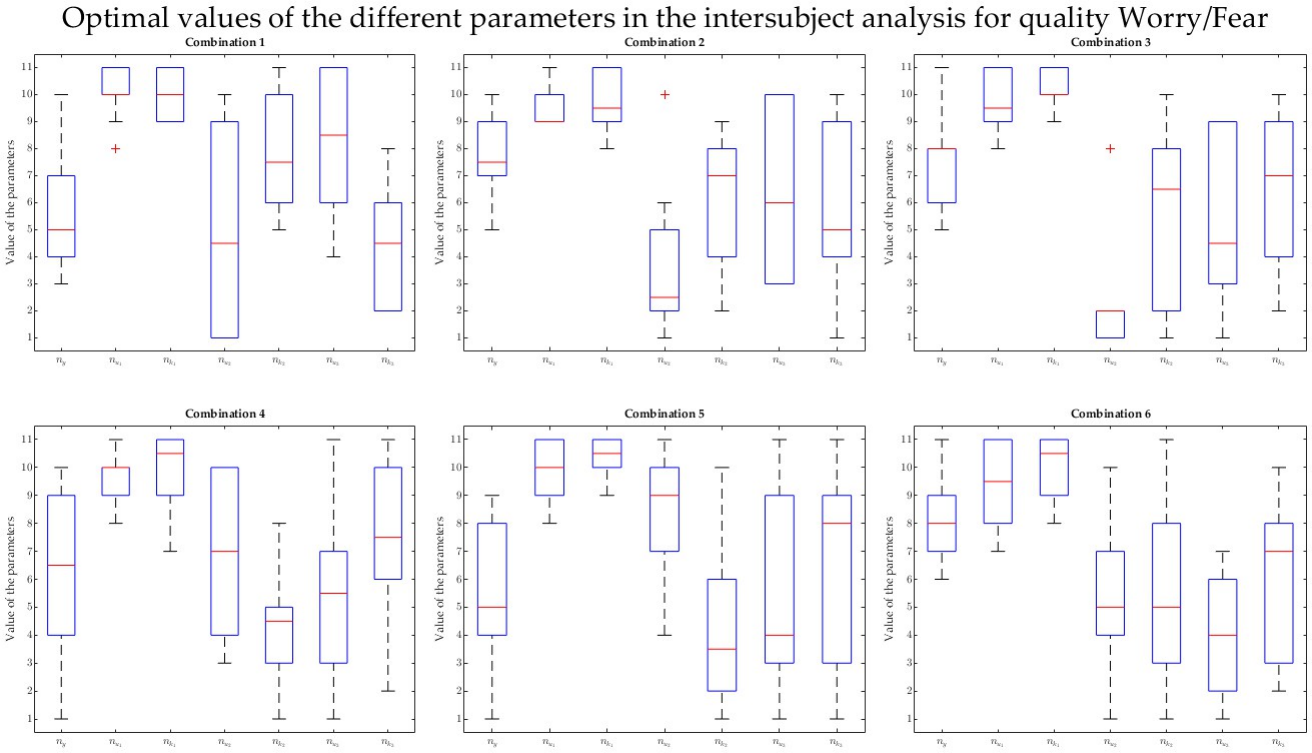
Boxplots of the values taken by the different parameters related to the intersubject models for Worry/Fear (Q3). The intensity prediction for Q3 considered regressors from emotion intensity *y* (*n*_*y*_) as well as from the features in the different combinations: combination 1—filtered HR ($n_{u_{1}}$), RSP rate ($n_{u_{2}}$) and expiration time ($n_{u_{3}}$); combination 2—filtered HR ($n_{u_{1}}$), RSP rate ($n_{u_{2}}$) and inhalation depth ($n_{u_{3}}$); combination 3—filtered HR ($n_{u_{1}}$), RSP rate ($n_{u_{2}}$) and exhalation depth ($n_{u_{3}}$); combination 4—filtered HR ($n_{u_{1}}$), expiration time ($n_{u_{2}}$) and inhalation depth ($n_{u_{3}}$); combination 5—filtered HR ($n_{u_{1}}$), expiration time ($n_{u_{2}}$) and exhalation depth ($n_{u_{3}}$); and combination 6—filtered HR ($n_{u_{1}}$), inhalation depth ($n_{u_{2}}$) and exhalation depth ($n_{u_{3}}$). The delays between each one of these physiological features and the emotion intensity are given by the respective $n_{k_{1}}$, $n_{k_{2}}$ and $n_{k_{3}}$.

A genetic algorithm was also used in this analysis to find the NARX parameters (related to the number of regressors and respective delays) that maximise the correlation between the predicted intensity and the ground-truth (reported intensity). Besides the correlations presented above, Tables 5–7 also contain the achieved parameters for each iteration, with the respective training and test sets. To assess whether these parameters were consistent or, instead, highly dependent on the set of subjects chosen for training and test, we obtained the boxplots represented in Figs 7, 8 and 9 for Q1, Q2 and Q3, respectively. Each one of these figures contains the distribution of the following parameters: number of regressors of the intensity *y* (*n*_*y*_), number of regressors of the physiological features *u*_*m*_ ($n_{u_{m}}$) and the delay of *u*_*m*_ with respect to *y* ($n_{k_{m}}$), with *m* up to the number of considered features (which was 1 for Q1, 2 for Q2 and 3 for Q3).

There are some observations regarding the boxplots that can be made. In general, it is possible to observe that, although most parameters are more spread, some of them are confined to a quite specific interval. For instance, the number of regressors related to filtered HR tends to be quite high for both Q1 and Q3, as it is possible to see in the boxplots of Figs 7 and 9, respectively. The same is true for the physiological delay of this feature. More specifically, using Q1 as an example, $n_{k_{1}}$ is never lower than 8, which means that this delay is always above 3.1 s. In the case of Q2, looking at how disperse the data in the boxplot is (Fig 8), all delays and regressors show a high variance and therefore it is not possible to establish them. It is not possible to understand the number of regressors related to the output emotion intensity *y* (*n*_*y*_), especially for Q1 and Q2 (their boxplots show a high variance).

### Common combinations of features

With the Apriori algorithm, we were able to assess which sets of features occurred often together to predict emotion intensity. As explained in Common combinations of features, this analysis had a maximum of 3 iterations, since we considered combinations of a maximum of 3 features for NARX models. This means that, in each iteration *k*, the algorithm analysed how many combinations of *k* features appear frequently enough. The results described here were obtained considering a threshold of 6 participants.

In the first iteration (no combination of features still to be detected), the following features appeared frequently using NARX models: filtered HR in Happiness/Joy (Q1); filtered GSR and expiration time in Disappointment/Regret (Q2); and filtered HR, RSP rate, inspiration time, expiration time, inhalation depth and exhalation depth in Worry/Fear (Q3). Regarding the results of the sliding-window LR, the frequent features were: GSR running rate, HR derivative and RSP derivative in Happiness/Joy (Q1); filtered GSR, HR derivative and expiration time in Disappointment/Regret (Q2); and finally filtered GSR and GSR derivative in Worry/Fear (Q3). Since this iteration considers the itemsets just containing one feature, the results could also be obtained by looking at the histograms of Fig 5.

In the second iteration, we found that no combination of 2 features was frequent enough (occurring in at least 6 participants) for any of the emotion qualities. This happened for both modelling approaches. Logically, there were also no frequent combinations of 3 features.

## Discussion

### Models for intensity estimation

The relevance of emotion intensity in experiencing an emotion has often been overlooked in emotion recognition studies, and much of the information we have about it comes from related conceptual studies and from the psychological perspective [34–36].

Despite this literature scarcity in intensity recognition, some researchers recognise the lack of including emotion intensity as an extra dimension to accurately recognise emotional states. For instance, some studies employ facial expressions to achieve this goal [37]. The work of Saxena *et al*. (2022) [21] proposed the recognition of emotion intensity levels (low, mild, and high) alongside valence and discrete emotions, from facial images using DL techniques. Saxena and colleagues acknowledged that, in spite of estimating intensity being challenging *per se*, the literature often overlooks emotion intensity due to a lack of databases in which this dimension is considered. To address this issue, they created their own database—“Facial Expression Intensity Levels Database” (FEILD), containing data from 13 subjects. Some features were extracted from landmarks, to perform intensity classification with diverse recognition algorithms such as support vector machines (SVMs), Naïve Bayes (NB) and convolutional neural networks (CNNs). Recently, speech has also been used, as in the case of Rajendran *et al*. (2023) [22] who differentiated between four levels of intensity (neutral, onset, offset, and apex) to be implemented in a voice-response system for assistive robots. The highest accuracy was obtained with a random forest (RF) algorithm.

While these studies applied static classification approaches, categorising intensity levels instead of estimating emotion intensity on a continuous scale, regression as performed in this work offers the advantage of capturing subtle variations. Regression-based methods provide the possibility to dynamically capture gradual shifts in the subjective feeling, leading to a more comprehensive understanding of emotional responses. In this sense, a regression approach facilitates the interpretation of results through CPM, since it is aligned with the idea of representing emotions as dynamic processes. Nonetheless, a robust and interpretable model is needed to ensure an adequate interpretation.

### Performance of NARX-based approach

With the use of NARX models, we managed to cover the need to apply a dynamic modelling approach to predict emotion intensity from inputs derived from different physiological signals for three distinct emotion qualities. In this study, we obtained a significant improvement in the intensity estimation when considering NARX models over a simple sliding-window LR, even if we limited the dynamic approach to a maximum of 3 features and this limitation was not applied to the sliding-window LR. The results of the individual dynamic models were in fact encouraging, with correlations varying between 0.539 and 0.866 for Happiness/Joy (Q1), between 0.477 and 0.786 for Disappointment/Regret (Q2), and between 0.492 and 0.823 for Worry/Fear (Q3). With the boxplots of the best subject-dependent correlations (Fig 4), we observed that these values were less disperse for NARX models than for sliding-window LR for all the qualities.

When considering the tables summarising the intrasubject models (Tables 2–4), one can observe the significant improvement when considering a multimodal approach for both modelling approaches (NARX and LR). This represents a major advantage over Barradas *et al*. (2022) [27], in which single physiological features were used at a time. Although these tables show that the best combinations of features differ from subject to subject, it is possible to locate some prominent features in the histograms with the feature ranking across subjects (Fig 5). Like all our analysis, these rankings were obtained separately for NARX models and the LR approach. When looking at the mentioned histograms, just Q2 presented similar relevant features for both modelling approaches, even in their ranking order. Interestingly, it is in Q1 that a feature is more prominent (filtered HR), which may indicate that time history is more important for this feature to predict intensity in case of Happiness/Joy. It is also possible to see that there is no relevant feature across the three qualities. Not finding common features across qualities led to the investigation of sets of features appearing frequently together in the intrasubject models, even if in a smaller set of subjects. However, we verified that no set of 2 or 3 features often appears together. In [23], Scherer reports the differences in the appraisal profiles for each quality, specifying the operation of the subsequent SECs. Since they are very different for each quality, they have distinct physiological effects. To a certain extent, this supports our choice of experimental design, in which we tried to maximise the differences between the induced qualities to best cover the different physiological responses.

Looking at the three qualities independently, some features were in fact predominant and thus, an intersubject analysis considering the most relevant features across subjects was explored. This intersubject modelling was made possible due to the dataset enlargement, when compared to Barradas *et al*. (2022) [27]. Once again, the dynamic approach significantly outperformed the sliding-window approach. In the intrasubject models, we could easily observe that both NARX models and the sliding-window LR benefitted from the addition of features. However, in the intersubject multimodal procedure, the sliding-window LR achieved very poor results (see, for instance, Fig 6 to see the dispersion of the two types of models for the 3 distinct qualities). As described, we used the most salient subjective-dependent features for training intersubject models; however, the observed intersubject correlations floating around 0 suggest that, when we used the sliding-window LR-model based on averaged windows for the intrasubject analysis, the dynamical effect was diminished. Therefore, the obtained relevant features are likely to be “stretched”, representing the average trial characteristics for each participant. This shows that the sliding-window approach is not suitable for problems with varying intensity. NARX models are also affected by intersubject variability, but the dynamic approach succeeded to find combinations of parameters (number of regressors and respective delays) that achieved high correlations (up to 0.642). Based on these results, we consider that the chosen NARX model could “extract” relevant features much better across subjects, benefitting the intersubject analysis. The best results of the intersubject analysis were obtained for Q3, the quality in which we considered the highest amount of features. This might indicate that the addition of features could benefit these models for Q1 and Q2, but we chose to just include features present in at least a quarter of the participants.

By considering the intersubject analysis, the number of regressors of the exogenous variable (*n*_*u*_) and the respective delay (*n*_*k*_) for Q1 were actually quite “concentrated” (see Fig 7). More specifically, filtered HR needed a large number of data points to contribute to the prediction of intensity (between 8 and 11) and its delay in relation to the intensity subjective feeling was high (between 9 and 11). It is not surprising that it is in Q1 that this consistency among the iterations considering different sets of testing and training sets occurs, since the appearance of this feature was the most predominant in the entire analysis. Observing the parameters of the intrasubject analysis (Table 5), they were also commonly high. Therefore, a lower dependency on the set of subjects were expected and confirmed. It is also interesting to report that the parameters related to filtered HR seem to “saturate” at the top of the plot, which may suggest that higher values would need to be tested. In the case of Q3, six combinations of parameters were tested, in which filtered HR was the only common feature among them. Curiously, it is also the only feature for which both the number of regressors and the physiological delay are consistently high across iterations, as it is possible to see in the second ($n_{u_{1}}$) and third ($n_{k_{1}}$) boxes of each boxplot of Fig 9. As in Q1, the parameters of this feature tend to “saturate” at the top of the plot.

When examining the predominant features for emotion intensity estimation for these two qualities (Q1 and Q3), a prevalence of the filtered HR is revealed, which is immediately followed by a set of distinct RSP-based features. This suggests a potential redundancy among some of these RSP-based features; in this sense, when combining them, we might be spreading their information over different features and reducing the models’ effectiveness. Therefore, by limiting our Q1- and Q3-models to be trained with filtered HR in combination with each predominant RSP-based features (separated models), we might identify a more suitable RSP-related feature for this combination. Also, such multimodal intersubject approach would help to determine whether filtered HR’s dominance over other features is still strong, or, in turn, if its interaction with individual RSP-features is more informative. Also, the two additional HR-related features rank very low, implying that filtered HR is indeed the key-feature to be combined in this analysis. In the case of Q2, filtered HR is among the 3rd ranked features, but the intensity estimation is predominated by the presence of GSR and RSP-related features. The same reasoning as before applies: we would need to test whether such features are redundant by applying the methodology just explained. As a final note on this matter, we mentioned earlier the saturation of filtered HR’s NARX parameters. Since it was a predominant feature for Q1 and Q3, as well as among the 3rd most important in Q2, there is the possibility of exploring higher number of regressors of this exogenous variable to be included in these models, for all qualities.

We found the results of the dynamic approach robust enough to fulfill our goal of interpreting them through the CPM, aiming for a clearer understanding of the emotional mechanisms for the considered qualities.

### CPM-based interpretation

The Scherer’s Component Process Model (CPM) [9] (see Introduction for more information) has been adopted in some emotion recognition studies. For instance, Somarathna *et al*. (2022) [18] considered different CPM components to predict different emotional states (particularly, emotion qualities). This study was pioneer because, with the same dataset, different appraisal components were used to train machine learning models. However, most components were based on subjective answers instead of more objective measures (like facial expressions, which they also recorded, but just correlated to the subjective answers). In the component related to physiological changes, they actually incorporated information from biosensors—which represents an advantage—but once again, this study only considered static features. In their study, 20 different qualities were classified and the intensity was disregarded. Contrarily, we analysed a smaller number of fixed qualities, but we are interested in understanding how the intensity is affected, with a model that predicts intensity over time instead.

In our study, the CPM is particularly relevant for interpretation purposes, since it considers the effects of the appraisal on other emotion components. As described earlier, the appraisal is built upon a set of sequential criteria (the “stimulus evaluation checks” or SECs) that influence all the emotion components (including the neurophysiological component), ending up in the integration of all those interactions in the form of the subjective feeling. Those SECs are, in chronological order, relevance (*how relevant is this event for me?*), implications (*what are the implications or consequences of this event?*), coping potential (*how well can I cope with or adjust to these consequences?*), and normative significance (*what is the significance of this event with respect to my self-concept and to social norms and values?*) [12]. Over the next paragraphs, we will discuss the effects of these SECs in the different emotional states analysed here, and whether this is witnessed in our results. We would like to emphasise the alignment between the emotion intensity estimation approach developed in this work with a CPM-based interpretation, given that the parameters of the NARX models give us greater clarity in the temporal contribution of different physiological features.

For Q1, the intrinsic pleasantness is high (due to the positive valence, related to the SEC of relevance). According to Scherer (2009) [11], this leads to changes in inhalation as well as to an HR deceleration. In the following SEC—implications—Happiness/Joy is perceived as highly goal conducive [23]. Following such appraisal, the literature reports a decrease in the RSP rate and a slight HR decrease [11]. This last physiological response is also shared by the following SEC—coping potential—since, for Q1, subjects feel in control and have a sense of power over the situation/event. However, this last SEC also causes an increase in the RSP rate. Given that 3 consecutive SECs cause changes in the HR, it is not unnatural that two features related to this physiological signal were predominant to predict intensity in the case of this emotion quality. Moreover, the analysis conducted to the number of regressors in the case of the intersubject models (Figs 7–9) shows that a high number of regressors is consistently needed in the case of HR ($n_{u_{1}}$), which is coherent with the fact that several SECs in a row affect this physiological feature.

In a 2D valence-control plan (see Geneva Emotion Wheel [19, 27]), Q2 differs from Q1 mainly in the valence axis, since it is a negative emotion. Negative valence, due to the assessment of intrinsic pleasantness of the stimulus under the SEC of relevance, can lead to increased SCR. Filtered GSR derivative (one of the most predominant features in this quality) is related to SCR. No literature was found describing the full appraisal profile of Disappointment/Regret and, thus, we do not infer other bodily responses caused by the different SECs.

Q3 is characterised by negative valence and low control. Negative valence, due to the assessment of intrinsic pleasantness of the stimulus under the SEC of relevance, can lead to increased SCR. Low control, under the SEC of coping potential, can cause a decrease in RSP rate and depth. Moreover, these two physiological features are also affected in the intermediate SEC—implications—if the subject considers the event obstructive to reaching their goals. In particular, there is an increase of SCR and also a faster and deeper RSP rate. Even though we do not have any information provided by the participants about their subjective experience with this SEC, the appraisal profile of fear included goal obstructiveness [9, 12]. In this sense, one can expect an increase of SCR in the first and second SECs, an increase of the RSP rate in the second SEC, and a decrease in the third SEC. The consistent appearance of physiological measures related to RSP rate and depth is aligned with the great dominance of RSP features for this quality. Even though one could expect a consistently high number of regressors regarding these features, our analysis showed that these parameters were dependent on the respective training/testing datasets. It is important to highlight that the second most predominant feature, inspiration time (present in 8 participants), was not computed for 6 participants, suggesting that this feature could have played an even more important role. Since it was disregarded in the intersubject analysis, we do not take any conclusions about the consistency of its regressors.

As presented in the Introduction, our choice of electrophysiological signals was motivated by their more reliable insights into internal states, since they are less prone to be masked [7, 38]—unlike facial expressions and speech. Nonetheless, these “external” features offer behavioural information that reflects emotional processes as well. Facial and vocal expressions represent, in fact, the motor expression component of the dynamic and recursive CPM [9, 11], which means that we could still record them and include information from this component, incorporate it into the models, and interpret it as done in this work. This could result into a more holistic understanding of emotional processes and, by extending dynamic emotion recognition models to incorporate all this information, open opportunities to enhance the robustness of systems for emotion recognition in real-life applications.

To summarise, the emotional response varies to some degree from subject to subject, as the results of the SECs depend subjectively on the individual’s perception of the event/stimulus [12]. This resulted in higher performance of intrasubject models compared to intersubject models. Nevertheless, obtained dynamical intersubject models reached a reasonable performance that has the potential to be further improved when considering the abovementioned suggestions and the avoidance redundant features. Besides, the architecture of dynamic NARX models provided a suitable foundation not just to achieve good results, but also to facilitate the interpretability of our results through the Scherer’s CPM. In this work, we collected real-time information related to two CPM components: the neurophysiological component (with data from three complementary peripheral physiological signals) and the subjective feeling (quality and intensity). With this, we managed to achieve a comprehensive framework to dissect dynamic emotional processes, and we introduced NARX models as a valuable modelling approach to analyse complex physiological interplays that might occur. Via the interpretation of the NARX parameters, we are able to get a deeper understanding of the dynamics inherent to emotions.

## Conclusion and future work

Affective computing is a rapidly-growing field, and yet, current methods for recognising and predicting emotions lack the reliability needed to be integrated into systems such as games, mental health monitoring and driver assistance technologies. One solution could be accounting for the dynamic nature of emotional processes, since they can lead to higher performances and better understanding of emotional processes. Our work contributes to the field with the development of a dynamic approach to predict emotion intensity recognition and prediction from combinations of peripheral physiological signals. The adopted method clearly outperformed a sliding-window linear regression, emphasising the importance of considering physiological variable’s history in the emotional process. Moreover, emotion intensity is one of the dimensions that defines the subjective feeling; hence, including this extra dimension in emotion recognition systems might enhance their performance. Unlike Cartesian emotion coordinates, intensity is intuitive for individuals, making the ground-truth more reliable. We therefore believe that estimating emotion intensity should play a role in real-world settings as the ones described at the beginning of this paragraph.

Specifically, we investigated nonlinear autoregressive exogenous models to predict output timeseries (emotion intensity *y*) by considering the time history of both output and exogenous inputs (physiological features *u*). We took advantage of the fact that NARX models support multiple-input, single-output nonlinear functions to develop a multimodal approach.

Current deep learning techniques (*e.g*., long short-term memory networks (LSTM) networks [39]) have also dynamic abilities and are able to achieve good results in predicting emotions if large enough training datasets are available. On the other hand, our proposed NARX models can not only be parameterised with much less data, but also come with the advantage of interpretability of model parameters in relation to appraisal theory. NARX models enable the assignment of a meaning to the parameters related to the number of regressors and to the delay between the inputs and the output variable.

Our approach went beyond Somarathna *et al*. (2022) [18] by examining emotion intensity and considering a dynamic modelling approach. For this, our experimental design with continuously measured subjective feeling represents a key advantage. Additionally, our modelling procedure combined physiological features as extra signals belonging to the CPM’s neurophysiological component to estimate emotion intensity—an improvement over Jenke & Peer (2018) [14] and Barradas *et al*. (2022) [27]. Our dataset was larger than these previous two works, allowing us to conduct an intersubject analysis.

The intersubject approach, that used the top-ranked physiological features as exogenous signals for each quality, revealed a notable dependency of some NARX parameters (number of regressors and delay) on the selected set of subjects for training and test. Besides, no sets of features occurring frequently together could be found. Such intersubject variability might be explained, to some extent, by the individual nature of emotional processes, that reflect personal needs, goals, and values, explaining to some extent also the obtained intersubject variability.

As discussed, certain physiological signals might be redundant, impacting the reliability and the access to physiological information in the context of emotion estimation. Although this represents a limitation of this work, we propose to combine filtered HR with individual RSP-based features for Q1 and Q3. Regarding Q2, two potential options emerge: either combining a single GSR-based feature with a single RSP-based feature, or adding filtered HR also as an input of the model. Lastly, for all qualities, a slightly higher number of HR-related regressors should be incorporated in the system, but keeping in mind that the model’s complexity increases rapidly with such increment. This requires a compromise between computational resources and performance.

Because of the explained complexity increase of NARX models, we downsampled our signals. As an alternative, the work of Jenke & Peer (2018) [14] could be extended to consider an architecture in which the different physiological signals, represented as distinct Dynamic Neural Fields (DNFs) [40], would interact among them. The stimulus evaluation checks would also need to be incorporated as a layer of this architecture, integrating all the information into the felt emotion intensity.

In the future, we plan to incorporate information from electroencephalogram in our model as part of another CPM’s component, since we also recorded this modality. Additionally, at the moment, we just consider non-interrupted influences of the physiological signals in the participants’ emotional state. However, the very same feature can be influenced by a certain SEC, no longer influenced by the following SEC, and again influenced by the SEC after (for instance), as seen in Scherer (2009) [11]. In this sense, we plan to superposition different NARX models representing different SECs for both intra and intersubject models. This will allow us to better interpret the role of physiological signals in emotion intensity.

Finally, we would like to recognise the importance of intensity to perceive and communicate emotions, and to highlight the potential for clinical applications. Regarding the latter, developed algorithms may be used to identify emotional disturbances which are characterised by maladjusted felt intensities of emotions that are either too intense or too weak [41].
